# Characterization of the R893C Na_V_1.5 mutation in Brugada syndrome

**DOI:** 10.3389/fcvm.2026.1726536

**Published:** 2026-02-19

**Authors:** Szabolcs Gaal, Beata Meszaros, Julianna Volko, Noemi Bilakovics, Muhammad Umair Naseem, Zoltán Pethő, Gabor Sandorfi, Gabor Menko, Orsolya Voros, Istvan Balogh, Gyorgy Panyi, Zoltan Csanadi, Tibor G. Szanto

**Affiliations:** 1 Department of Biophysics and Cell Biology, Faculty of Medicine, University of Debrecen, Debrecen, Hungary; 2 Division of Cardiology, Department of Cardiology, Faculty of Medicine, University of Debrecen, Debrecen, Hungary; 3 Doctoral School of Molecular Medicine, Debrecen, Hungary; 4 Institute of Physiology II, University of Münster, Münster, Germany; 5 Department of Human Genetics, Faculty of Medicine, University of Debrecen, Debrecen, Hungary

**Keywords:** arginine, Brugada syndrome, DTT reagent, gating, loss-of-function

## Abstract

Brugada syndrome (BrS) is a genetically determined cardiac arrhythmogenic syndrome with increased risk of sudden cardiac death. BrS is mostly caused by mutations in *SCN5A* gene encoding the primary ɑ-subunit of the cardiac sodium channel Na_V_1.5. We aimed at characterizing the functional alterations caused by the *R893C* mutation, identified in a proband diagnosed with BrS, and establishing whether the mutation is associated with BrS. Although several mutations have been reported in the close vicinity of *R893*, the functional role of this region remains unknown and, in addition, exploring *SCN5A* mutations in patients with inherited arrhythmogenic syndromes is critical for understanding the pathogenesis of arrhythmias. The mutations were introduced by site-directed mutagenesis. The variants were transiently expressed in CHO cells and potassium currents were measured using the whole-cell patch clamp technique. Patch clamp recordings have demonstrated that *R893C* almost completely abolished the sodium current, I_Na_, though the mutation did not exert dominant-negative effect on wild-type Na_V_1.5 channels. We also observed significant decrease in channel activation and a depolarized shift of steady–state inactivation curve, however, the kinetics of inactivation and recovery from fast inactivation were not changed by the mutation. Moreover, the reducing agent Dithiotreitol partially restored the normal function of Na_V_1.5 in the *R893C* mutant highlighting a likely mechanism for loss of conduction via formation of disulphide bridges. We showed that *R893H* channels also failed to produce any detectable I_Na_ that confirms the importance of the highly conserved *R893* in gating. Our study reveals *R893C* is a loss-of-function mutation with altered electrophysiological characteristics of Na_V_1.5. Thus, *R893C* may contribute to the BrS phenotype of the proband. Our findings may facilitate the understanding of the mechanisms of arrhythmogenesis in BrS, as it helps to identify mutational hotspots in BrS. Moreover, our work may improve novel gene therapy and new therapeutic drug design targeting Na_V_1.5 channelopathies.

## 1 Introduction

Na_V_1.5 plays a key role in the generation of action potential in cardiomyocytes; therefore, its normal function is essential for a normal heart rhythm. Consequently, alterations of the sodium current (I_Na_), conducted by Na_V_1.5 in the human heart, may lead to diseases responsible for cardiac arrhythmias, such as the Brugada Syndrome (BrS), which is a potentially lethal, heritable arrhythmia syndrome. BrS, first described in 1992, is characterized by an ST segment elevation in the right precordial leads of the 12-lead electrocardiogram (ECG) and an increased risk for sudden cardiac death (SCD) due to ventricular fibrillation (VF), without major structural alterations in the heart (1, 2). The prevalence of BrS is in the range of 1–5 in every 10,000 individuals, and approximately 4% of all SCD cases and 20% of all unexplained sudden deaths with structurally normal heart are related to BrS (3). More than ten different genes have been associated with BrS (3, 4), but the most demonstrated one is *SCN5A*, a gene encoding the *α* subunit of Na_V_1.5. Approximately 10%–30% of subjects with BrS carry an *SCN5A* gene mutation with autosomal dominant inheritance (1, 5). The *α* subunit of the Na_V_1.5 protein is composed of 2016 amino acids and consists of four homologous domains (designated as DI-DIV) forming a pseudo tetrameric structure. Each domain contains six transmembrane helical segments (S1–S6) linked by intra- and extracellular loops. Of these, segments S1−S4 from each domain form the voltage sensor domain (VSD) that regulates channel opening upon depolarization. Segments S5, S6, and the connecting extracellular pore-loops (P-loops) of domains DI to DIV form the channel pore and the selectivity filter (SF). To date, more than 300 *SCN5A* mutations affecting different structural parts of Na_V_1.5 have been identified in BrS patients, most of them are loss-of-function (LOF) mutations resulting in either reduced channel expression in the plasma membrane, current density, or alterations in gating kinetics of Na_V_1.5, such as the voltage dependence of activation or inactivation (6–8). To identify and characterize novel Na_V_1.5 mutations in patients with BrS is critical for a better understanding of the arrhythmogenesis including the structural-functional relationship of the Na_v_1.5 during gating transitions, which may promote the invention of new and more efficacious drug therapy for Na_V_1.5 channelopathies. Further, the exploration of the relationship between a specific mutation and the clinical presentation may provide practical and helpful information for risk stratification in a disease with strikingly variable clinical course and prognosis (9–12).

**Figure 1:**
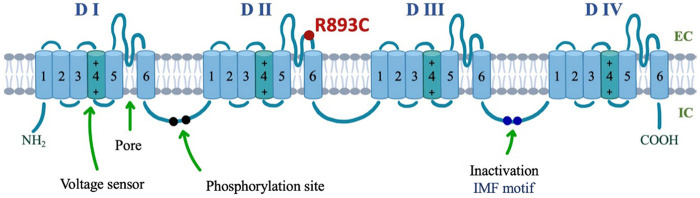
Location of R893C in the topological structure of the *α*-subunit of Na_v_1.5. The *α*-subunit is a long polypeptide folding into four domains (DI–DIV), each consisting of six transmembrane spanning regions (S1–S6). S1–S4 comprises the voltage-sensing domain (VSD) with the highly positively charged S4 being the main component (depicted in a darker shade). S5 and S6 comprising the pore-forming domain. The inactivation gate is found on the intracellular linker between DIII and DIV and is composed of an IFM (isoleucine, phenylalanine, methionine) motif. Location of R893C mutation in the predicted topologic structure of the Na_V_1.5, marked by a red circle.

Herein, we report a study that characterizes a missense *R893C* mutation in Nav1.5. Position 893 is in the highly conserved pore region (S5-S6) in domain II of Na_V_1.5 (Figure 1). The mutation was associated with the clinical phenotype of BrS in a patient diagnosed previously by the Cardiology department at our university (13). The proband is a 41-year-old man referred for urgent coronary intervention because of a recent onset sharp chest pain. The clinical course of the patient has been described in detail (13), herein, we summarize the most relevant features. The chest pain was considered atypical for acute coronary syndrome, and the levels of electrolytes and cardiac biomarkers were repeatedly within the normal range. The 12-lead trans telephonic electrocardiogram (TTECG) sent by the emergency team from the patient's home demonstrated coved ST-segment elevation >2 mm, followed by negative T waves in leads V1-V2, corresponding to Brugada type I ECG pattern (Supplementary Figure S1A). These abnormalities, however, were resolved spontaneously and a normal ECG was recorded upon arrival at the hospital (Supplementary Figure S1B). Transthoracic echocardiography demonstrated normal left ventricular function with no wall motion abnormality. Aorta dissection and pulmonary embolism were excluded by cardiac CT angiography. Further diagnostic workup focused on BrS. Polymorphic ventricular tachycardias were induced with both programmed ventricular stimulation and with the administration of 400 mg intravenous procainamide (Supplementary Figure S2). In both instances, the arrhythmia terminated spontaneously within one minute and the patient remained responsive during the episodes. The patient's history also showed multiple episodes of loss of consciousness with seizure since the age of 16, which was attributed to epilepsy, although the results of repeated electroencephalographic (EEG) exams were normal. Antiepileptic therapy with different medications failed to prevent the convulsive attacks which occurred during the night usually 5 to 10 times annually, throughout the past 25 years. As the diagnosis of Brugada syndrome was established, a dual-chamber implantable cardioverter-defibrillator (ICD) was implanted. At this point, we considered the possibility that the nighttime seizure attacks, or at least some of them could have been the consequence of VTs with spontaneous termination related to BrS. However, several seizure spells occurred during a more than 10-year follow up after the ICD implantation, and the ECG recovered from device memory showed normal sinus rhythm or sinus tachycardia during all these episodes. Moreover, repeated EEG monitoring with 24-hour sleep deprivation eventually provided diagnostic findings: the electrical signals recorded during a habitual seizure together with the clinical presentation were suggestive of the autosomal dominant nocturnal frontal lobe epilepsy.

Based on the decrease in the current density of cells expressing the *R893C* mutant and reported by (12, 14), the mutation was classified as pathogenic. However, the detailed biophysical analysis of the gating parameters of the Na_V_ current of the *R893C* mutant were not revealed in the previous reports, and the potential of its dominant-negative effect was not elucidated either. The analysis of the *R893C* mutation presented here extends our knowledge from its clinical manifestations to detailed biophysical characterization and modelling.

## 2 Methods

### 2.1 Ethics statement

This study was approved by the local Ethics Committee of the University of Debrecen (Debrecen, Hungary) and conforms with the principles outlined in the Declaration of Helsinki. Blood samples were obtained after participants signed a written informed consent. The collection and patients' involvement in the study were recruited between 01/04/2018 and 01/04/2019.

### 2.2 Genetic analysis

Genetical analysis was performed via whole genome sequencing, which was performed by Novogene (Cambridge, UK). Data analysis was done using GATK pipeline. The detected possible causative variant (*SCN5A R893C*) was confirmed using Sanger sequencing by the BigDye Terminator v3.1 Cycle Sequencing kit (Applied Biosystems, Foster City, CA, USA) according to the manufacturer's protocol. Primer sequences were: 5'-tcccctcctcttcctccttc-3' and 5'-gatggatggacggatgggta-3'.

### 2.3 Site-directed mutagenesis

Wild-type (WT) human *SCN5A* (Uniprot reference: Q14524-1) was subcloned and GFP-tagged (at N-terminal) into pcDNA3.1/Zeo(+) vector previously (kindly provided by H. Abriel, University of Bern, Switzerland). Both *R893C*- and *R893H*-Na_v_1.5 mutations were created by site-directed mutagenesis using overlap extension PCR. *R893C* mutation was carried out by UD-GenoMed Kft. (Debrecen, Hungary), the mutant clone was confirmed by direct sequencing. *R893H* mutation was performed using the following primers (mutation underlined):

Na_V_1.5_Flank F 5' GTGAATTCGAGGAGATGCTGCAG 3'

Na_V_1.5_Flank R 5' CAGGTACCCGGCCCC 3'

Na_V_1.5_Mut F 5‘ CATCATCTTCCACATCCTCTGTG 3’

Na_V_1.5_Mut R 5' CACAGAGGATGTGGAAGATGATG 3'

The mutant DNA fragment was substituted in the WT-Na_v_1.5_GFP_ coding plasmid using EcoRI and KpnI restriction enzymes. The mutant clone was directly sequenced to verify the presence of the desired mutation and the absence of additional variations.

### 2.4 Cell culture and transfection

Chinese hamster ovary (CHO) and human embryonic kidney (HEK) cells were cultured in Dulbecco's modified Eagle's medium (DMEM, Gibco, Cat# 11965084)) containing 10% fetal bovine serum (FBS, Sigma-Aldrich), 2 mM L-glutamine and 100 µg/mL streptomycin and 100 U/mL penicillin-g (Sigma-Aldrich) at 37 °C in 5% CO_2%_ and 95% air humidified atmosphere and were passaged twice per week following a 2–3 min incubation in trypsin-EDTA (Invitro-gen). Cells were transiently transfected with 1 µg of the plasmid containing either the wild type human Na_V_1.5 ion channel gene tagged with a GFP at the N-terminus (WT-Na_V_1.5_GFP_), or the same construct containing *R893C* mutation (*R893C*-Na_V_1.5_GFP_) or with the human wild type Na_V_1.5 lacking the GFP tag (WT-Na_V_1.5, a kind gift from F. Bosmans, Ghent University, Gent, Belgium), using Lipofectamine 2,000 kit (Invitrogen, Carlsbad, CA), as described by the manufacturer's protocol. Transfected cells were trypsinized with trypsin-EDTA allowing the effective re-suspension of the cell cultures, washed twice with 2 mL of ECS (see below), and seeded onto 35-mm polystyrene cell culture dishes (Cellstar, Greiner Bio-One) to a density that enabled single cells to be identified. GFP-positive transfectants were identified with a Nikon Eclipse TS100 fluorescence microscope (Nikon, Tokyo, Japan) using bandpass filters of 455–495 nm and 515–555 nm for excitation and emission, respectively, and were used for current recordings (>70% success rate for co-transfection). Ionic currents were typically recorded 24 to 36 h after transfection.

### 2.5 Confocal microscopy

CHO cells were plated one day before the transfection on 8-well chambered coverslips (7,000/well, ibidi GmbH, Gräfelfing, Germany). 0.4 µL FuGene HD (Promega, Madison, WI) was mixed with 120 or 140 ng DNA (per well) in serum- and phenol red-free Opti-MEM (Thermo Fisher Scientific, Waltham, MA) and added to the cells. The cells were labeµLled with Alexa Fluor ™ 647 NHS (succinimidyl) ester dissolved in 1 × TBS (Tris-buffered saline; 25 mM Tris–HCl, 150 mM NaCl, pH = 7.5) for 20 min on ice before the measurements. Confocal microscopic measurements were carried out at room temperature 48 h after labelling. Confocal images were taken using an A1 confocal microscope (Nikon, Tokyo, Japan). Based on the fluorescent intensity of the GFP and Alexa Fluor™ 647 NHS Ester signals, the overlapping part of the signals was determined by using the Intensity Plot Profile tool of the ImageJ software. The intensity values were normalized to the maximum intensity.

### 2.6 Detection of GFP-tagged ion channels protein in the membrane fraction

Cells were transiently transfected with WT-Na_V_1.5_GFP_ or *R893C*-Na_V_1.5_GFP_ encoding gene, and 48 h later the membrane proteins were extracted from the cells using Subcellular Protein Fractionation Kit for Cultured Cells (ThermoScientific, 78840) according to manufacturer's protocol. Proteins from the membrane fraction were separated using 6% SDS–polyacrylamide gel. Na_V_1.5 channel proteins together with the GFP were detected using Alpha Innotech gel documentation system. The protein extraction was confirmed using Coomassie blue staining.

### 2.7 Cellular electrophysiology

Whole-cell Na^+^ (I_Na_) currents were recorded by patch-clamp recordings with patch pipettes pulled from GC150F-7.5 borosilicate capillaries (Harvard Apparatus Co., Holliston, MA) with tip diameters between 0.5 and 1 μm and heat polished to a tip resistance ranging typically 2–8 M*Ω*. The micropipettes were pulled in four stages by using a Flaming Brown automatic pipette puller (Sutter Instruments, San Rafael, CA). I_Na_ recordings were carried out using an Axopatch 200B amplifier (Axon Instruments, Foster City, CA, USA), connected to personal computers using Digidata 1550A data acquisition hardware (Molecular Devices Inc., Sunnyvale, CA). In general, the holding potential was −120 mV and series resistance errors were reduced by approximately 70%–80% with electronic compensation. The cutoff threshold for acceptable series resistance values was 15 MOhm. Records were discarded when leak at the holding potential was more than 10% of the peak current at the given test potential. Online leak correction was used with P/4 leak subtraction. The sampling frequency was set accordingly to the desired voltage protocol (see below). The liquid junction potential (LJP) between ECS and ICS solutions (see below) was estimated as −8.9 mV using the built-in function of pClamp and was also experimentally measured according to Neher (15). The measured LJP was −9.1 ± 0.1 mV (*n* = 4). The V_m_ values reported in the manuscript are values without LJP correction. Thus, the real membrane potential is the reported Vm−∼9 mV. We have used the same extra- and intracellular solutions for the biophysical characterization of all constructs, therefore, the same LJP applies to all records. Experiments were performed at room temperature ranging between 20 and 24 °C. Data were analyzed using the pClamp10 software package (Molecular Devices Inc., Sunnyvale, CA) and GraphPad Prism 8 (GraphPad, CA). Before analysis, current traces were digitally filtered with a three-point boxcar smoothing filter.

### 2.8 Solutions

For I_Na_ recordings the extracellular (bath) solution (ECS) contained 145 mM NaCl, 5 mM KCl, 2.5 mM CaCl_2_, 1 mM MgCl_2_, 10 mM [4-(2-hydroxyethyl)-1-piperazineethanesulfonic acid] (HEPES), and 5.5 mM glucose (pH = 7.35 titrated with NaOH). To avoid the recordings of endogenous K^+^ currents a Cs ^+^ -based intracellular (pipette) solution (ICS) was used, thus, the ICS consisted of (in mM) 10 NaCl, 105 CsF, 10 HEPES, and 10 [ethylene glycol-bis(*β*-aminoethyl ether)-N,N,N′, N′-tetraacetic acid] (EGTA) (pH = 7.2 titrated with CsOH). The osmolarity of ECS and ICS were between 302 and 308 mOsm/L and ∼295 mOsm/L, respectively. Bath perfusion around the measured cell with different extracellular solutions was achieved using a gravity flow micro perfusion system at a rate of 200 uL/min. Excess fluid was removed continuously. Solutions containing pharmacology agents, e.g., Tetrodotoxin (TTX, Sigma-Aldrich), 1,4-Dithiotreitol (DTT, Sigma-Aldrich), or procainamide (Vifor Pharma España, S.L.) were made fresh in ECS from stocks. Stock solutions were prepared from powder dissolved in de-ionized water. The effect of the tested compounds at a given concentration was characterized as the normalized current calculated as I/I_0_, where I_0_ and I are the peak currents in the absence and presence of the compound at the indicated concentration, respectively. A choline-based ECS was used as a perfusion control, its composition was (in mM) 145 Choline-Cl, 5 KCl, 10 HEPES, 5.5 glucose, 2.5 CaCl_2_, and 1 MgCl_2_. The prominent change in the current amplitude upon perfusion with the choline-based solution was an indicator of both the successful expression of the Na_V_1.5 constructs and the proper operation of the perfusion system.

### 2.9 Voltage protocols

To determine the biophysical properties of the Na_V_1.5 variants, we studied the kinetics of activation, fast inactivation, the kinetics of recovery from fast inactivation, and the voltage dependence of steady-state activation and inactivation. To study activation and fast inactivation kinetics, cells were depolarized to 0 mV for 15 ms from a holding potential of −120 mV every 5 s (sampling frequency was 20 kHz). To avoid the model dependence of the reported parameter characterizing Na_V_ channel activation kinetics, we assessed the time-to-peak variable (16). Time-to-peak was measured as the time between the initiation of the depolarizing pulse and the time point when I_Na_ reached the peak. The average of the time-to-peak for a particular cell was determined as the average of time-to-peak values obtained for 4–5 depolarizing pulses to 0 mV repeated at every 5 s. The current densities in pA/pF for a particular cell were defined as the ratio of the average of peak currents, obtained for at least three depolarizing pulses to the appropriate test potentials repeated at every 5 s in a sequence, to the cell capacitance. The rapid activation of the current is followed by a decay corresponding to fast inactivation of Na_V_1.5. To obtain the time constant of inactivation (*τ*_i_) a single exponential function was fitted to the decaying part of the current traces $I(t)=I_{0}e^{\left(-t/\tau_{i}\right)}+C$, where I_0_ is the amplitude of the inactivating component of the current, *τ*_i_ is the inactivation time constant, and C is the steady-state current at the end of the depolarizing pulse. To determine the voltage dependence of steady-state activation of the Na^+^ current, the cells were held at −120 mV and depolarized for 100 ms to test potentials ranging from −70 to +50 mV in 10-mV increments every 5 s (sampling frequency was 50 kHz). Peak conductance [G(V)] at each test potential was calculated using $G(V)=I_{\;peak}/(V_{m}-V_{Na})$, where I_peak_ is the peak current at a test potential of V_m_ and V_Na_ is the reversal potential of Na^+^ (+68.7 mV, based on the Nernst equation using the ionic composition of ECS and ICS). The G(V) values were then normalized for the maximum conductance (G/G_0_) and plotted as a function of test potential along with the best-fit Boltzmann function $G/G_{0}=1/(1+e^{-(V_{m}-V_{1/2})/s})$, where V_m_ is the actual test potential, V_½_ is the midpoint voltage, and s is the slope factor of the function. To describe the voltage dependence of steady-state inactivation (termed as h∞), the fraction of non-inactivated channels at each test potential was calculated as I/I_−120_, where I is the peak current evoked by a test pulse to 0 mV for 15 ms from a given prepulse potential, and I_−120_ is the peak current evoked by the test pulse from the holding potential of −120 mV. The duration of the time interval between the test pulses was 5 s. V_1/2_ and s were determined by fitting a Boltzmann function to the data points. To evaluate the late I_Na_, we analyzed the steady-state Na^+^ current during the last 5 ms of the 15-ms-long depolarizing pulses to 0 mV i.e., between 10 and 15 ms. TTX-sensitive late Na^+^ current was defined as the magnitude of the steady-state current in the presence of 0.5 µM TTX and subtracted this value from the current measured in the absence of TTX in the same time-window. To study the kinetics of recovery from inactivation, pairs of depolarizing pulses were applied from the holding potential of −120 to 0 mV for 100 ms (sampling frequency was 20 kHz). The duration of the first step was used to inactivate channels and measure initial peak current amplitude (I_1_). After a recovery period, defined as the interpulse interval (ipi) at −120 mV, the second identical voltage step was applied, and peak amplitude of the recovered current (I_2_) was measured. The interpulse interval (ipi) at −120 mV varied between 0.25 and 60 ms. The fractional recovery (FR) at a given ipi was calculated as FR = (I_2_)/(I_1_). The FR vs. ipi plot was fit with an exponential function rise to a maximum containing a single term, $FR(ipi)=1-e^{\left(-ipi/\tau_{r}\right)}$ to give the time constant of recovery from inactivation, *τ*_r_. Prior to analysis, current traces were corrected for ohmic leak. Nonlinear least squares fits were done using the Levenberg-Marquardt algorithm. For all data acquisition the filter frequency (8 pole Bessel filter) was set to half of the sampling frequency, according to the Nyquist theorem.

### 2.10 Protein structure modeling

Structural modeling was performed using the cryo-EM structure of the human Na_V_1.5 [PDB: 7DTC (17)]. Conserved regions, including regions around the *R893C* mutant, are fully resolved in this structure with 3.30 Å resolution. Based on the crystal structure, *R893* was mutated to cysteine with UCSF Chimera X (18), followed by a protonation and an energy minimization step (GAFF force field). Putative ligand interactions were plotted with LigPlot (19).

### 2.11 Statistical analysis

The number of experiments on different cells (n) involved in the given analysis is shown in the text. The number of independent transfections/biological replicates (N) was at least 4 in most sets of experiments. Data are presented as means ± SEM, individual data points are indicated in the bar graphs. Statistical significance was estimated with GraphPad Prism 8 software (version 8.0.1, La Jolla, CA). The Shapiro–Wilk test was used to test for normality of the given set of data. For pairwise comparisons Student's t test or Mann–Whitney rank sum test (nonnormal distribution population) was applied. For multiple comparisons one-way ANOVA with *post hoc* Tukey's test was applied. Statistical significance is indicated in terms of *P* values. However, when *P* < 0.0001 was reported, our statistical program was unable to determine the exact *P* value. In these cases, we indicated *P* < 0.0001.

### 2.12 Limitations

The number of cells investigated in our study was different for the different voltage protocols and pharmacological experiments. Most cells had to be excluded from the data analysis regarding the determination of the time constant of activation and inactivation. However, the number of these cells did not exceed 10, either. In the case of pharmacological measurements, the number of these cells was negligible (only 1–2 cells had to be eliminated).

## 3 Results

### 3.1 Family pedigree and identification of *R893C* Na_V_1.5 channel mutation

**Figure 2:**
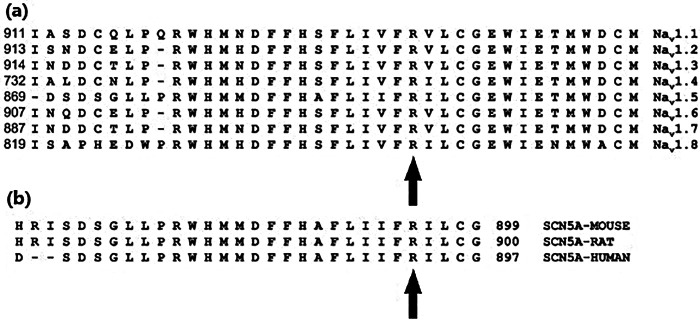
Sequence alignment of the DII S5-S6 region of Na_V_ channel isoforms in the close vicinity of residue 893 in Na_V_1.5 (up arrows). R893 residue showed a high degree of conservation in human Na_V_ channel isoforms **(a)** and in different species (mouse, rat and human) **(b).**

Mutation screening via next generation whole genome sequencing identified a single heterozygous nucleotide transition (C-to-T) at position 2,677 of the *SCN5A* gene (c.2677C > T), leading to the missense mutation in which an arginine, R, was replaced by cysteine, C, at position *893* (*R893C*). Algorithms developed to predict the effect of missense changes on protein structure and function (SIFT, PolyPhen-2, Align-GVGD) all suggested that this variant is likely to disrupt Nav channel function *SCN5A 893*C was absent in a large cohort (6,500) of healthy individuals (6), and was not found in the Human Gene Mutation Database (HGMD) (20), Ensembl, HapMap, and 1,000 genomes project. Thus, *R893C* in Na_v_1.5 may be responsible for BrS in harmony with previous findings (6, 21). Position *893* is in the extracellular linker between S5 and S6 transmembrane segments in domain II (DII) of the Na_v_1.5 protein (Figure 1) and located in the pore near the selectivity filter. Although the precise role of *R893* in Na_V_1.5 is unknown, the presence of and arginine at this position has been evolutionary favored. Comparison of aligned amino acid sequences demonstrates that *R893* is highly conserved among different human Na_v_ channel isoforms (Figure 2A). Furthermore, it is also highly conserved among *SCN5A* in different species including mice, rat and human (Figure 2B).

**Figure 3:**
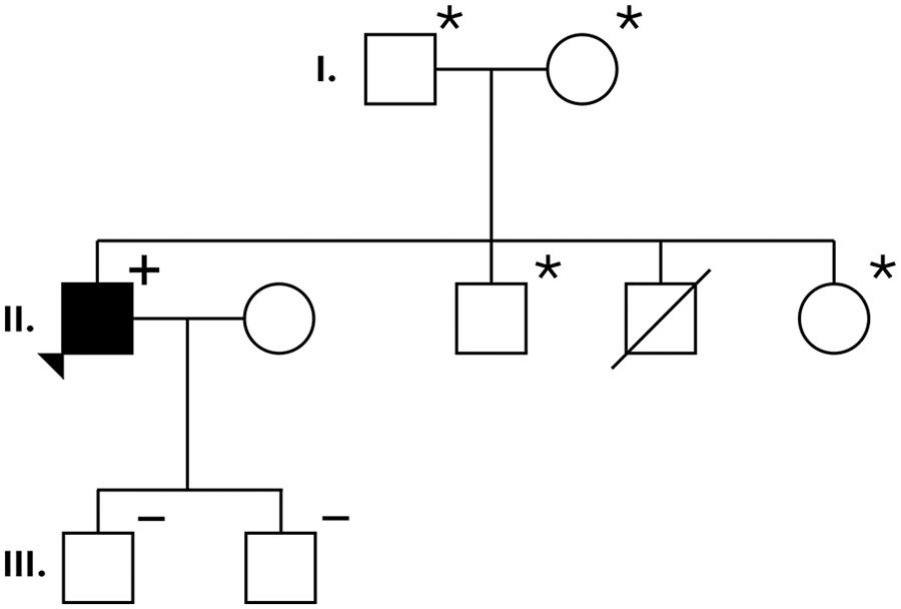
Family pedigree with phenotypic and genotypic information. Males are shown as squares, females are shown as circles. Symbol “+” marks the variation carrier for SCN5A c.2677C > T. The proband was a male member with BrS, indicated by an arrowhead pointing to a filled square. Diagonally crossed symbol indicates a deceased family member. Symbol “–” marks family members not carrying the mutation. Symbol “*” marks family members whose genome sequence was not analysed.

The proband has 2 sons (III-1 and III-2, Figure 3), both are completely asymptomatic with no signs of structural heart disease during transthoracic echocardiography. One of them (III-1) showed normal pattern, the other one (III-2) had an incomplete right bundle branch block (RBBB) on the resting 12-lead ECG. No genetical abnormality was found in any of them and both refused invasive EP study. Although one of the proband's brother died at his early age, but he died due to consequences of hepatocellular carcinoma and not by cardiac arrhythmia.

### 3.2 Biophysical characterization of WT-Na_V_1.5_GFP_

First, we tested the effect of the GFP-tag located at the N-terminus of Na_V_1.5 (WT-Na_V_1.5_GFP_) by comparing the gating characteristics to wild-type Na_V_1.5 (WT-Na_V_1.5). Sodium currents were recorded in CHO cells in whole-cell configuration and the essential gating parameters were determined including the voltage-dependence of steady-state activation and steady-state inactivation, the kinetics of activation, inactivation, and recovery from inactivation. The currents were fully activated by 15-ms-long depolarizing pulses to 0 mV for both WT-Na_V_1.5 and WT-Na_V_1.5_GFP_ (Supplementary Figure S3A). CHO cells transfected with plasmid for WT-Na_V_1.5 showed average peak current densities of 311.8 ± 31.8 pA/pF (*n* = 15) (Supplementary Figure S3B). Similar currents were observed at a mean peak level of 340.6 ± 36.2 pA/pF (*n* = 31) for WT-Na_V_1.5_GFP_, showing no significant difference compared to WT-Na_V_1.5 (*P* = 0.6150) (Supplementary Figure S3B and Table 1). The activation kinetics showed a small, but biologically not relevant difference: the time-to-peak values were 0.413 ± 0.019 ms (*n* = 15) for WT-Na_V_1.5 and 0.493 ± 0.018 (*n* = 31) for WT-Na_V_1.5_GFP_ (*P* = 0.008) (Supplementary Figure S3B). Time constants of fast inactivation (*τ*_i_) were obtained by fitting decaying exponential functions to the current traces recorded during 15-ms-long depolarizing pulses to 0 mV (Supplementary Figure S3A). *τ*_i_ for WT-Na_V_1.5 and WT-Na_V_1.5_GFP_ were 0.719 ± 0.065 ms (*n* = 15) and 0.869 ± 0.047 ms (*n* = 26), respectively (Supplementary Figure S3B, Table 1) the difference was not statistically significant (*P* = 0.0658). The G-V curve was calculated from the current-voltage relationship, which was obtained using a series of 100-ms-long depolarizing pulses ranging from −70 mV to +50 mV in 10-mV increments every 5 s. The voltage dependence of the conductance was determined by fitting a Boltzmann function to the normalized conductance (G/G_0_)-test potential relationships to give the midpoint voltage (V_1/2_) and slope factor (s) (Supplementary Figure S3C; see Methods for details). We found that the GFP tag does not modify either the midpoint voltage or the slope factor of the function for Na_V_1.5, as the midpoint voltages were V_1/2_ = –30.1 ± 2.4 mV and s = 5.8 ± 0.4 mV (*n* = 6) for WT-Na_V_1.5 and V_1/2_ = –34.2 ± 1.8 mV and s = 6.1 ± 0.3 mV (*n* = 23) for WT-Na_V_1.5_GFP_ (*P* = 0.2846 and *P* = 0.6591 for V_1/2_ and s, respectively) (Supplementary Figure S3C, inset). To assess the voltage-dependence of steady-state inactivation, we applied 5-s-long prepulse potentials from −120 to −10 mV in 10-mV increments before stepping the test potential to 0 mV for 15 ms. The fraction of non-inactivated channels at each voltage was calculated as I/I_−120_ and plotted as a function of prepulse potential and the voltage dependence of steady-state inactivation was characterized by V_1/2_ and a slope factor determined from Boltzmann fits (see Materials and Methods) (Supplementary Figure S3D). The GFP tag did not cause a shift in the h∞ curves: the V_1/2_ values were V_1/2_ = –54.2 ± 2.9 mV and s = –8.4 ± 0.5 mV (*n* = 10) for WT-Na_V_1.5 and −59.1 ± 2.1 mV and s = –9.5 ± 0.5 mV (*n* = 12) for WT-Na_V_1.5_GFP_ (*P* = 0.1676 for V_1/2_ and *P* = 0.1461 for s), respectively (Supplementary Figure S3D, inset). The kinetics of recovery from inactivation were studied using depolarizing pulse pairs and varying the interpulse interval (ipi) between the depolarization steps (see Materials and Methods for details). The two steps voltage protocol and a representative I_Na_ recording for characterizing the kinetics of recovery WT-Na_V_1.5_GFP_ are shown in Supplementary Figure S4. The recovered fraction (FR) of the channels vs. ipi plot was fit with a single-exponential function to give the time constant of recovery from inactivation (*τ*_r_) (Supplementary Figure S3 E). *τ*_r_ for WT-Na_V_1.5 and WT-Na_V_1.5_GFP_ were 3.20 ± 0.40 (*n* = 9) and 3.40 ± 0.20 (*n* = 22) ms, respectively (*P* = 0.6283) (Supplementary Figure S3E, inset). The essential gating parameters of WT-Na_V_1.5 and WT-Na_V_1.5_GFP_ are summarized in Table 2.

**Table 1:** Current densities of Na_V_1.5 constructs expressed in CHO cells.

	Current density	
	pA/pF	*N*
WT-Na_V_1.5	311.8 ± 31.8 (*P* = 0.6150)	15
WT-Na_V_1.5_GFP_	340.6 ± 36.2	31
R893C-Na_V_1.5_GFP_	15.8 ± 2.2 (*P* < 0.0001)	19
Mixed	373.4 ± 33.9 (*P* = 0.7560)	47
R893H-Na_V_1.5_GFP_	17.3 ± 3.5 (*P* < 0.0001)	28

Values are expressed as mean ± SEM. *P* values are indicated vs. WT-Na_V_1.5_GFP_ group.

**Table 2:** Gating parameters of Na_V_1.5 constructs expressed in CHO cells.

Variant	Activation kinetics		Inactivation kinetics		Voltage-dependence of steady-state activation			Voltage-dependence of steady-state inactivation			RFI	
	time to peak at 0 mV (ms)	*n*	*τ*_i_ at 0 mV (ms)	*n*	V_1/2_ (mV)	s (mV)	*n*	V_1/2_ (mV)	s (mV)	*n*	*τ*_r_ (ms)	*n*
WT-Na_V_1.5	0.413 ± 0.019 (*P* = 0.008)	15	0.719 ± 0.065 (*P* = 0.0658)	15	−30.1 ± 2.4 (*P* = 0.2846)	5.8 ± 0.4 (*P* = 0.6591)	6	−54.2 ± 2.9 (*P* = 0.1676)	−8.4 ± 0.5 (*P* = 0.1461)	10	3.2 ± 0.40 (*P* = 0.6283)	9
WT-Na_V_1.5_GFP_	0.493 ± 0.018	31	0.869 ± 0.047	26	−34.2 ± 1.8	6.1 ± 0.3	23	−59.1 ± 2.1	−9.5 ± 0.5	12	3.4 ± 0.20	22
*R893C*-Na_V_1.5_GFP_	0.779 ± 0.079 (*P* < 0.0001)	20	0.933 ± 0.085 (*P* = 0.4892)	20	−24.9 ± 2.1 (*P* = 0.0033)	8.9 ± 0.7 (*P* = 0.0002)	12	−47.4 ± 3.0 (*P* = 0.0037)	−11.3 ± 0.7 (*P* = 0.0455	10	3.65 ± 0.36 (*P* = 0.4798)	12
Mixed	0.532 ± 0.013 (*P* = 0.0515)	46	1.082 ± 0.045 (*P* = 0.0457)	47	−29.4 ± 1.5 (*P* = 0.0520)	7.6 ± 0.3 (*P* = 0.0047)	44	−52.1 ± 2.2 (*P* = 0.0578)	−9.7 ± 0.5 (*P* = 0.7522)	27	3.90 ± 0.19 (*P* = 0.1020)	45

The kinetics of activation was obtained from time to peak at 0 mV are indicated in the Activation kinetics column. The inactivation time constants (τ_i_) obtained from single-exponential fits and the time constants for the fast components of the decay are indicated in the Inactivation kinetics column. V_1/2_ and s values were obtained by the Boltzmann function to the data. τ_r_ is the time constant for recovery from inactivation (RFI), n indicates the number of independent experiments. See the Materials and methods for the equations used for fitting. Statistical significance against WT-Na_V_1.5_GFP_ is indicated.

### 3.3 *R893C* dramatically decreases peak I_Na_ and modifies Na_V_1.5 channel activation kinetics

**Figure 4:**
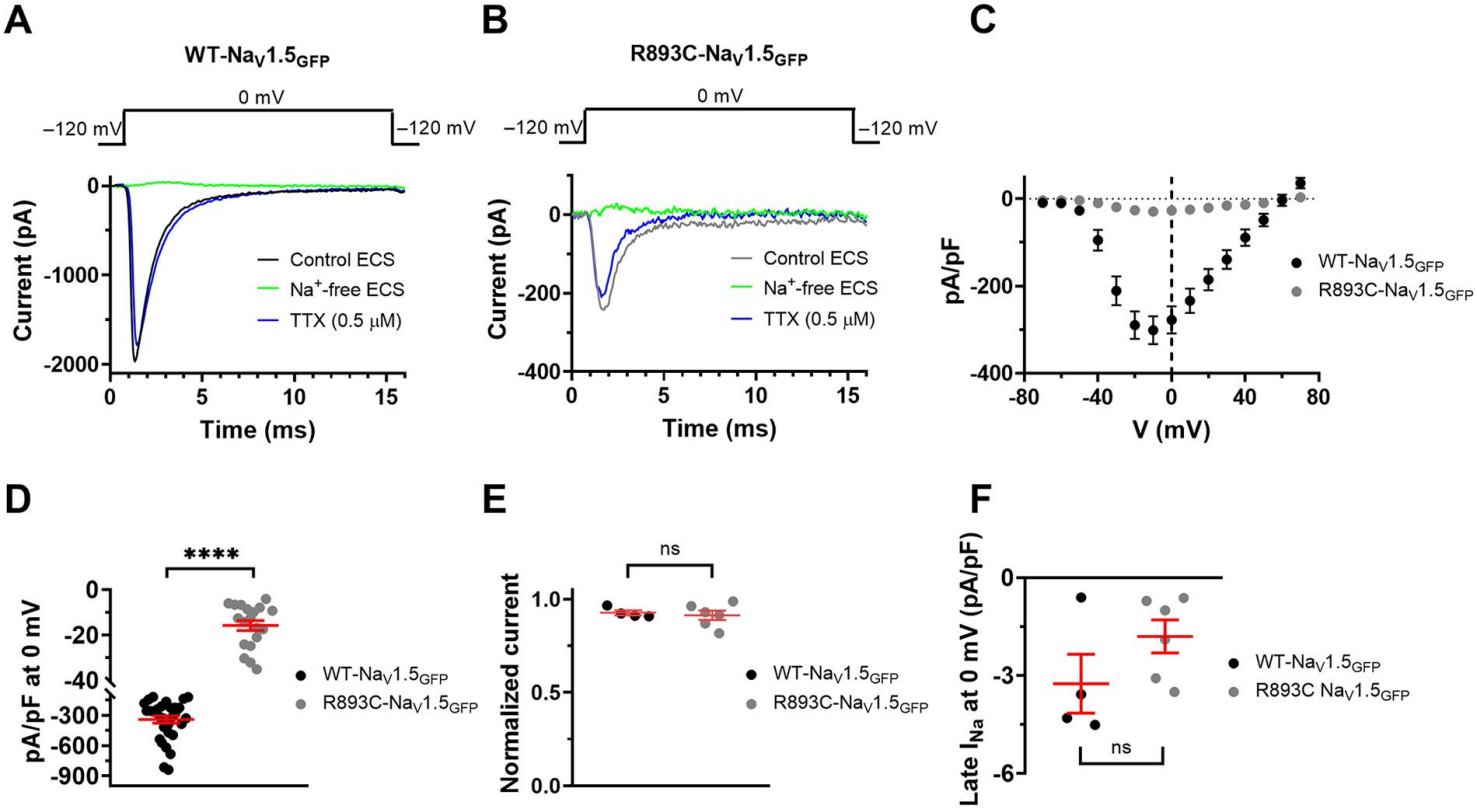
R893C dramatically decreases the current density of Na_V_1.5. Representative whole-cell current traces were recorded for WT- **(A)** and R893C-Na_V_1.5_GFP_ channels **(B)** expressed in CHO cells. The voltage protocol is shown above the raw current traces, pulses were delivered every 5 s in control conditions (WT – black, R893C—gray), in the absence of extracellular Na^+^ (Na ^+^ -free ECS, green), and in the presence of 0.5 µM Tetrodotoxin (TTX, blue). **(C)** Current densities measured at different test potentials. Cells were held at −120 mV and depolarized to gradually increasing test potentials ranging from −70 to +70 mV in steps of 10 mV for 15 ms every 15 s before returning to the holding potential. I_Na_ amplitude was normalized to the cell capacitance to obtain current density values at each test potential for WT- (black) and R893C-Na_V_1.5_GFP_ (gray). **(D)** current densities measured at 0 mV. **(E)** Remaining current fraction (RCF) was calculated as I/I_0_ where I and I_0_ are the peak current at 0 mV in the presence and absence of 0.5 µM TTX at equilibrium block, respectively. **(F)** Current densities of late I_Na_ recorded at 0 mV. Symbols indicate individual data points obtained for WT- (black) and R893C-Na_V_1.5_GFP_ (gray). Values are expressed as mean ± SEM. Differences were considered significant (****, when *P* < 0.0001, t-test) compared to WT-Na_V_1.5_GFP_).

A heterozygous variation at position 2,677 of the *SCN5A* gene (c.2677 C > T) was identified in the proband's DNA that leads to an arginine to cysteine substitution at position *893* (p.*R893C*) of the Na_V_1.5 protein (*R893C*-Na_V_1.5_GFP_). The mutation was proposed earlier to contribute to the BrS phenotype (12, 14); thus, we conducted patch-clamp studies to assess the effect of the mutation *R893C* in whole cell configuration. Cells, transfected with *R893C*-Na_V_1.5_GFP_ coding plasmid, were clamped at a holding potential of −120 mV and subjected to 15 ms duration depolarization pulses to 0 mV at pulse frequency of 0.2 Hz. Current traces in Figures 4A,B show that I_Na_ is dramatically reduced by the *R893C* mutation. The peak Na^+^ current density values, calculated at different test potentials for the *R893C*-Na_V_1.5_GFP_, were smaller than in WT-Na_V_1.5_GFP_ transfected cells (Figure 4C, Table 1). Figure 4D shows the current densities determined at 0 mV that were (in pA/pF) 340.6 ± 36.2 (*n* = 31) for WT-Na_V_1.5_GFP_ and 15.8 ± 2.2 (*n* = 19) for *R893C*-Na_V_1.5_GFP_, respectively (*P* < 0.0001). This may mean that the *R893C* mutation causes gross structural changes in the channel thereby disrupting Na^+^ conduction, gating, or channel trafficking/stability and/or the combination of these.

We recorded very small Na^+^ currents in our experimental system in non-transfected CHO cells (current density at 0 mV is 4.6 ± 1.2 pA/pF, *n* = 15, Supplementary Figure S5). To eliminate the possibility that the recorded Na^+^ currents upon transfection with *R893C*-Na_V_1.5_GFP_ are endogenous currents, we tested the effect of 0.5 µM tetrodotoxin (TTX) applied extracellularly, since endogenous Na^+^ channels are sensitive to 0.5 µM TTX [I_Na_ in CHO cells could be fully eliminated after treatment with 0.5 µM TTX (22)], whilst the cardiac Na_V_1.5 is not (23, 24). Macroscopic currents were recorded in the continuous presence of 0.5 µM TTX (dissolved freshly in the ECS, see Materials and Methods) in WT- and *R893C*-Na_V_1.5_GFP_ transfected cells at 0 mV every 5 s (Figures 4A,B). The currents recorded in the presence or absence of TTX were virtually overlapping for both WT-Na_V_1.5_GFP_ and *R893C*-Na_V_1.5_GFP_ transfected cells upon ∼3 min continuous perfusion of the cells with TTX. The normalized current, calculated as I/I_0_, where I_0_ is the peak current in the absence, and I is the peak current at equilibrium block by 0.5 µM TTX), was 0.927 ± 0.013 (*n* = 4) and 0.913 ± 0.025 (*n* = 6) for WT-Na_V_1.5_GFP_ and *R893C*-Na_V_1.5_GFP_, respectively (*P* = 0.7029) (Figure 4E). We also determined the current densities of late I_Na_ as the TTX sensitive current. To do this, we measured the magnitude of the steady-state current (average of the last 5 ms of the depolarizing steps) in the presence of 0.5 µM TTX and subtracted this value from the current measured in the absence of TTX in the same time-window. The TTX sensitive current then was expressed in pA/pF. The values were 3.3 ± 0.9 (*n* = 4) and 1.8 ± 0.5 (*n* = 6) for WT-Na_V_1.5_GFP_ and *R893C*-Na_V_1.5_GFP_, respectively (*P* = 0.1680) (Figure 4F). Moreover, additional experiments were performed in HEK cells to exclude the possibility that our observations, i.e., the reduced current density of *R893C*-Na_V_1.5_GFP_ were specific to the CHO expression system. The Na^+^ current density at 0 mV was even smaller when *R893C*-Na_V_1.5_GFP_ was expressed in HEK (5.4 ± 0.7 pA/pF, *n* = 9, Supplementary Figure S5) as compared to CHO.

To address whether the decreased current density in *R893C*-Na_V_1.5_GFP_ transfected cells is a consequence of biogenesis/trafficking deficiency, we used confocal microscopy and SDS-page analysis. In confocal microscopy experiments we took advantage of the GFP tag on the N-termini of WT-Na_V_1.5_GFP_ and *R893C*-Na_V_1.5_GFP_ channels. The plasma membrane localization of the GFP-tagged constructs was assessed by comparing the GFP fluorescence signal to the Alexa Fluor™ 647 NHS Ester fluorescence intensity emitted from the plasma membrane. The normalized pixel-by-pixel intensity profile analysis of WT-Na_V_1.5_GFP_ and *R893C*-Na_V_1.5_GFP_ transfected cells confirm the same GFP expression pattern for both WT-Na_V_1.5_GFP_ and *R893C*-Na_V_1.5_GFP_ (Supplementary Figure S6). These results suggest persistent trafficking and expression of mutant channel *R893C*-Na_V_1.5_GFP_ to the plasma membrane. Plasma membrane expression of the WT-Na_V_1.5_GFP_ or *R893C*-Na_V_1.5_GFP_ proteins was also confirmed by the comparison of the GPF signal of the proteins extracted from the membrane fractions of the transiently transfected cells (Supplementary Figure S7). The protein extraction was verified using Coomassie blue staining of the same SDS–polyacrylamide gel. Supplementary Figure S7 indicates the expression of Na_V_1.5 constructs in the membrane in comparable amount. The negative control was the extract of native i.e., non-transfected CHO cells for which, therefore, no GFP signal was detected (Supplementary Figure S7). Taking together all these findings, we suggest that the loss of Na^+^ current resulting from the *R893C* mutation is caused by the loss of function of Na_v_1.5 channels, rather than altered trafficking of otherwise functional channels.

**Figure 5:**
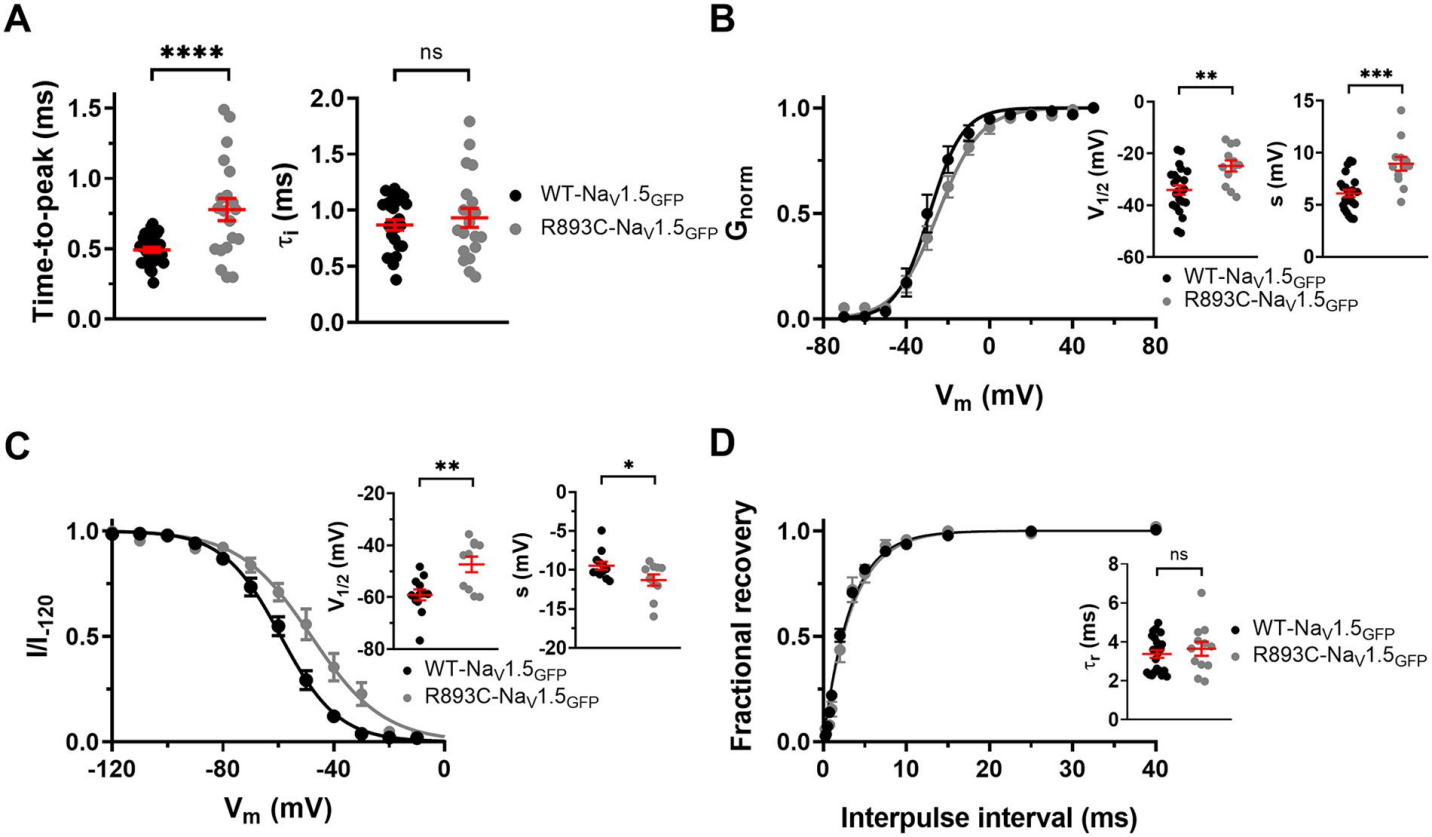
Biophysical characterization of R893C-Na_V_1.5_GFP_. Identical voltage protocols were used for WT-Na_V_1.5_GFP_ (black) and R893C-Na_V_1.5_GFP_ (gray) throughout the Figure. **(A)** To study activation and inactivation kinetics, cells were repeatedly depolarized to 0 mV for 15 ms from a holding potential of −120 mV. Kinetics of activation was characterized by determining the time-to-peak of I_Na_ shown in Figures 4A, B. Inactivation time constant (*τ*_i_) was determined by fitting a single exponential function to the decaying part of the currents shown in Figure 2. **(B)** The voltage-dependence of steady-state activation was determined from the peak currents (I_peak_) at test potentials V_m_ ranging from −70 mV to +60 mV in 10-mV increments every 5 s using G**(V)**=I_peak_/(V_m_–V_Na_), where V_Na_ is the reversal (Nernst) potential of Na ^+^ . The G**(V)** values were normalized for the maximum conductance (G_norm_) and plotted as a function of test potential. The superimposed solid lines show the best fit Boltzmann function. Points indicate individual data points of the midpoint voltages (V_1/2_) of steady-state activation. **(C)** Steady-state inactivation was determined by depolarizing the cells at 0 mV for 15 ms from a holding potential of −120 mV to elicit sodium currents (I_–120_) and then applying a series of 5 s conditioning prepulse potentials ranging from −120 mV to 0 mV in 10 mV increments, with each voltage step followed by a constant 15 ms test pulse to 0 mV to determine I. Sodium currents recorded at each prepulse potential were normalized to their respective maximal values (I/I_–120_), averaged, and plotted as a function of the prepulse potential. Data points indicate individual data points of the midpoint voltages (V_1/2_) of steady-state inactivation. **(D)** Kinetics of recovery from inactivation was studied using a two-pulse protocol with increasing interpulse time, (ipi, from 0.5 to 40 ms) between the pulses. Data points presented as fractional recovery (FR) and plotted as a function of ipi. The time constant of recovery (t_r_) was determined by fitting the averaged data points with a single exponential function, the superimposed solid lines show the best fits. Symbols indicate individual data points. Error bars indicate the mean ± SEM of the investigated parameter throughout the Figure. *, **, ***, and **** indicate significant differences (t-test) when *P* < 0.05, *P* < 0.01, *P* < 0.001, and *P* < 0.0001, respectively.

In addition to the reduction in current density for the *R893C*Na_V_1.5_GFP_ construct (Figure 4B), the mutation confers altered kinetic properties to WT-Na_V_1.5_GFP_, as well (Table 2). Figure 5 shows the essential biophysical parameters of *R893C*-Na_V_1.5_GFP_ as compared to WT-Na_V_1.5_GFP_. The kinetics of activation and inactivation were determined using 15-ms-long depolarizing pulses to 0 mV every 5 s. To describe the activation kinetics, we determined the time-to-peak variable (see Methods for details). We found that *R893C* has significant effect on channel activation: time-to-peak is 0.493 ± 0.018 ms (*n* = 31) and 0.779 ± 0.079 ms (*n* = 20) for WT- and *R893C*-Na_V_1.5_GFP_, respectively (*P* < 0.0001) (Figure 5A). The inactivation time constant of the currents were determined by fitting a single exponential function to the decaying part of the currents shown in Figures 4A,B. In contrast to the kinetics of activation, we did not observe significant change in the inactivation kinetics; *τ*_i_ was (in ms) 0.869 ± 0.05 (*n* = 26) and 0.933 ± 0.08 (*n* = 20) for WT- and *R893C*-Na_V_1.5_GFP_, respectively (*P* = 0.4892) (Figure 5A). We have also studied the voltage dependence of the activation kinetics in a range between −30 to +30 mV in 10-mV steps (Supplementary Figure S8). The activation kinetics of the current is faster with increasing depolarizations, and the time constants are greater (i.e., kinetics is slower) for the *R893C*-Na_V_1.5_GFP_ at each test potential. Moreover, we found that the *R893C*-Na_V_1.5_GFP_ displays a ∼10 mV positive shift in the voltage-dependence of the steady-state activation toward more positive potentials together with a significant increase in the slope factor (V_1/2_ = –34.2 ± 1.8 mV and s = 6.1 ± 0.3 mV, *n* = 23, for WT-Na_V_1.5_GFP_ and V_1/2_ = –24.9 ± 2.1 mV and s = 8.9 ± 0.7 mV, *n* = 12 for *R893C*-Na_V_1.5_GFP_; *P* = 0.0033 and 0.0002 for V_1/2_ and s, respectively) (Figure 5B). Similarly, the voltage-dependence of steady-state inactivation was shifted by ∼10 mV to more depolarized potentials with a shallower slope for the *R893C*-Na_V_1.5_GFP_ construct (V_1/2_ = –59.1 ± 2.1 mV and s = –9.5 ± 0.5 mV, *n* = 12, for WT-Na_V_1.5_GFP_; V_1/2_ = –47.4 ± 3.0 mV and s = –11.3 ± 0.7 mV, *n* = 10 for *R893C*-Na_V_1.5_GFP_; *P* = 0.0037 and *P* = 0.0455 for V_1/2_ and s, respectively) (Figure 5C). We also compared the kinetics of recovery from inactivation induced by a 100-ms depolarization to 0 mV, followed by a variable recovery interval interpulse (ipi) at −120 mV (the voltage protocol is shown in Supplementary Figure S4). The kinetics of recovery from inactivation for both WT- and *R893C*-Na_V_1.5_GFP_ were well-fit using a single-exponential component. Our data showed that the *τ*_r_ in *R893C*-Na_V_1.5_GFP_ is not different from that of the WT-Na_V_1.5_GFP_ channels (Figure 5D); *τ*_r_ was (in ms) 3.38 ± 0.19 (*n* = 22) and 3.65 ± 0.36 (*n* = 12) for WT-Na_V_1.5_GFP_ and *R893C*-Na_V_1.5_GFP_, respectively (*P* = 0.4798).

### 3.4 The *R893C*-Na_V_1.5_GFP_ mutant channels do not exert a dominant negative effect on WT- Na_V_1.5_GFP_

**Figure 6:**
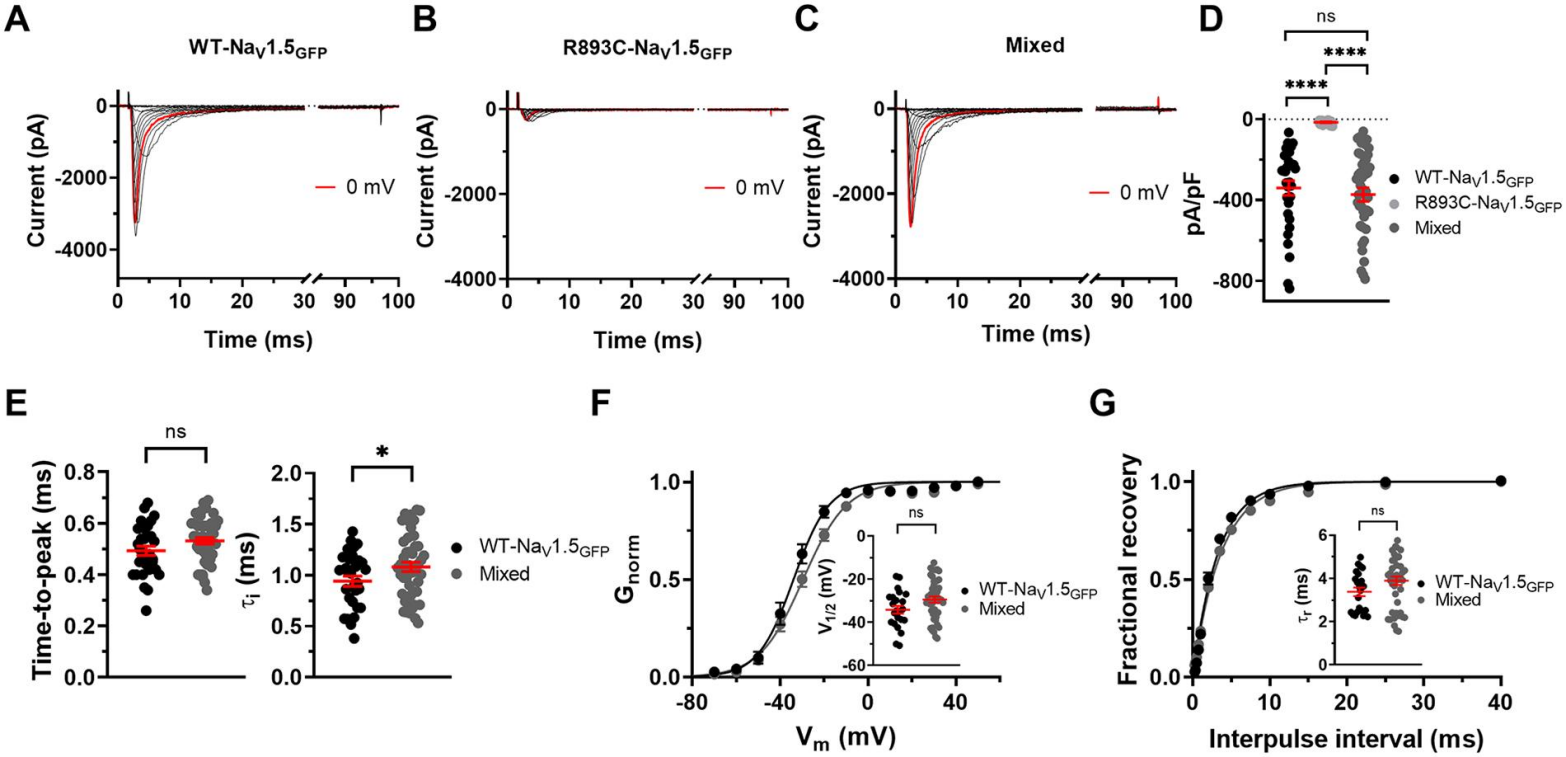
Electrophysiological characterization of WT-Na_V_1.5_GF*P*_ + R893C-Na_V_1.5_GFP_ heteromer channels (mixed). Representative whole cell Na^+^ current traces were recorded in cells transfected with of 1 µg of WT **(A)**, 1 µg of R893C **(B)**, and Na_V_1.5_GFP_ + R893C-Na_V_1.5_GFP_ plasmids at 1:1 molar ratio using 1-1 ug DNA from both plasmids **(C)** recorded at different test potentials ranging from −80 mV to +100 mV in 10-mV increments from a holding potential of −120 mV. Currents recorded at 0 mV are highlighted with red. **(D)** Peak current densities at 0 mV for WT-Na_V_1.5_GFP_ (black), R893C-Na_V_1.5_GFP_ (light gray) channels alone and co-expressing them together at 1:1 molar ratio (Mixed, dark gray). **(E)** Rise time to maximum Na ^+^ -current amplitude (time-to-peak, left) and time constant of inactivation (*τ*_i_, right) at 0 mV for WT-Na_V_1.5_GFP_ (black) and Mixed channels (dark gray). **(F)** The voltage dependence of steady-state activation was determined from currents recorded at different test potentials. Normalized curves were fitted to the average data points using Boltzmann function. Inset shows the midpoint voltage for WT-Na_V_1.5_GFP_ (black) and WT + R893C-Na_V_1.5_GFP_ (dark gray). **(G)** The assessment of fractional recovery from inactivation was performed with the voltage protocol described in Materials and methods. Time constants of recovery are displayed for WT-Na_V_1.5_GFP_ and WT-Na_V_1.5_GFP_ + R893C-Na_V_1.5_GFP_ channels, respectively. Results are expressed as mean ± SEM; symbols indicate individual data points obtained for the different Na_V_1.5 constructs throughout the Figure. Differences were considered significant (****, when *P* < 0.0001, t-test, except panel D where ANOVA was used (see Materials and Methods 2.11).

To test whether *R893C* has a dominant-negative effect on WT channels, we recorded I_Na_ in CHO cells co-transfected with the WT-Na_V_1.5_GFP_ and *R893C*-Na_V_1.5_GFP_ channels at 1:1 ratio (later termed as Mixed) **(**Figures 6A–C**)**. We maintained the concentration each construct the same as in single-transfection experiments (section 3.3) i.e., 1 µg WT and 1 µg *R893C* plasmids were used in the co-transfection experiments. In this scenario, any dominant-negative effect of the *R893C*-Na_V_1.5_GFP_ construct should be reflected in a reduction of the peak I_Na_ as compared to WT-Na_V_1.5_GFP_ single-transfection data. We found that the trafficking competent but gating-defective *R893C* mutant does not exert a dominant-negative effect, as we did not observe a significant reduction in the peak current densities compared to the WT. The current densities at 0 mV were (in pA/pF) 340.6 ± 36.2 (*n* = 31) for WT-Na_V_1.5_GFP_ and 373.4 ± 33.9 (*n* = 47) for Mixed channels, respectively (*P* = 0.7560) (Table 1 and Figure 6D). In cells co-expressing WT and *R893C* channels there was no shift of the V_1/2_ of activation and inactivation compared with WT expressed alone (WT: V_1/2_ = –34.2 ± 1.8 and s = 6.1 ± 0.3 mV, *n* = 23; Mixed: V_1/2_ = –29.4 ± 1.5, s = 7.6 ± 0.3 mV, *n* = 44; *P* = 0.0520 for V_1/2_ and *P* = 0.0047 for s for steady-state activation; WT: V_1/2_ = –59.1 ± 2.1 and s = –9.5 ± 0.5 mV, *n* = 12; Mixed: V_1/2_ = –52.1 ± 2.2 and s = –9.7 ± 0.5 mV, *n* = 27; *P* = 0.0578 for V_1/2_ and *P* = 0.7522 for s, for steady state inactivation) (Figure 6F**,** Table 2). Similarly, we did not observe significant differences in the activation kinetics of the currents (WT: time-to-peak=0.493 ± 0.018 ms, *n* = 31; Mixed: time-to-peak = 0.532 ± 0.013, *n* = 46; *P* = 0.0515) (Figure 6E**,** Table 2). The kinetics of recovery from inactivation were also similar for the WT and Mixed records: *τ*_r_ were (in ms) 3.38 ± 0.20 (*n* = 22) and 3.90 ± 0.19 (*n* = 45) (*P* = 0.1020) for WT and Mixed channels, respectively (Figure 6G**,** Table 2). On the other hand, a slight but statistically significant increase was observed in the time constant of fast inactivation when cells expressed the Mixed population of channels as compared to WT single transfected ones. The *τ*_i_ were (in ms) 0.869 ± 0.05 ms (*n* = 26) and 1.082 ± 0.05 (*n* = 47) (*P* = 0.0457); for WT and Mixed channels, respectively (Figure 6E**,** Table 2).

### 3.5 Procainamide inhibits the Na_V_1.5 regardless of the R893C mutation

**Figure 7:**
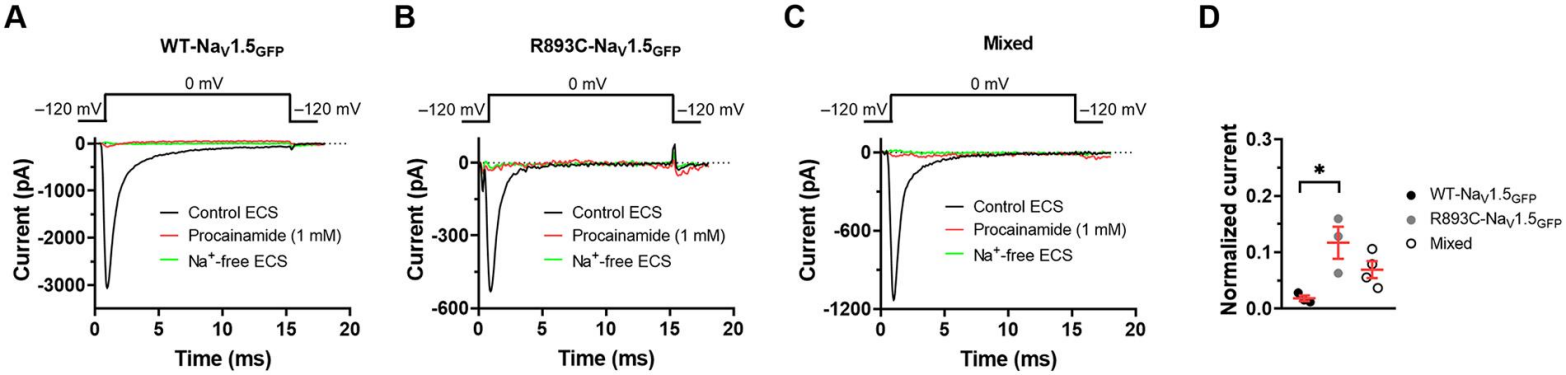
Effect of 1 mM procainamide applied extracellularly on Na_V_1.5 currents. Whole-cell current traces were recorded for WT-Na_V_1.5_GFP_ **(A)**, R893C-Na_V_1.5_GFP_ **(B)**, and Mixed **(C)** constructs expressed in CHO cells. The voltage protocol is shown above the raw current traces; pulses to 0 mV were delivered every 5 s in control conditions (Control ECS, black), in the absence of extracellular Na + (Na ^+^ -free ECS, green), and in the presence of 1 mM procainamide (red). **(D)** Normalized current was calculated as I/I_0_, where I and I_0_ is the peak current at 0 mV in the presence and absence of 1 mM procainamide at equilibrium, respectively. Symbols indicate individual data points for WT- (black), R893C-Na_V_1.5_GFP_ (gray), and WT-Na_V_1.5_GFP_ + R893C-Na_V_1.5_GFP_ (Mixed, empty circles) channels, respectively. Values are expressed as mean ± SEM. Significant differences among groups (ANOVA) were identified (*P* < 0.05), * indicates significant difference (Tukey's *post hoc*).

BrS was triggered in the proband by procainamide administration (Supplementary Figure S2) (13). Therefore, the sustained sensitivity of the mutant current to procainamide had to be demonstrated. The procainamide sensitivity of the WT current is in Figure 7A. The current was eliminated upon perfusion of the bath solution with 1 mM procainamide (25, 26). Figure 7B shows a similar phenomenon for the R893C-Na_V_1.5_GFP_. The current is eliminated, the record obtained in the presence of procainamide overlaps with the one obtained in Na ^+^ -free extracellular solution. We also took advantage of the experimental layout in section 3.4, i.e., we repeated the experiments for mixed channel as well, where WT-Na_V_1.5_GFP_ and R893C-Na_V_1.5_GFP_ were co-expressed to mimic the heterozygous state of the patient. The inhibition of I_Na_ current was also complete in the presence of procainamide for the “Mixed” channels (Figure 7C). The quantitative analysis in Figure 7D shows that the remaining fraction of the peak currents is less than 0.12 in the presence of 1 mM procainamide, regardless of the constructs transfected; normalized currents were 0.018 ± 0.005 (*n* = 3), 0.117 ± 0.028 (*n* = 3, *P* = 0.0198), 0.069 ± 0.015 (*n* = 4, *P* = 0.1796) for WT-Na_V_1.5_GFP_, R893C-Na_V_1.5_GFP_, and Mixed channels, respectively.

### 3.6 Dithiotreitol treatment increases the *R893C*-Na_V_1.5_GFP_ current

**Figure 8:**
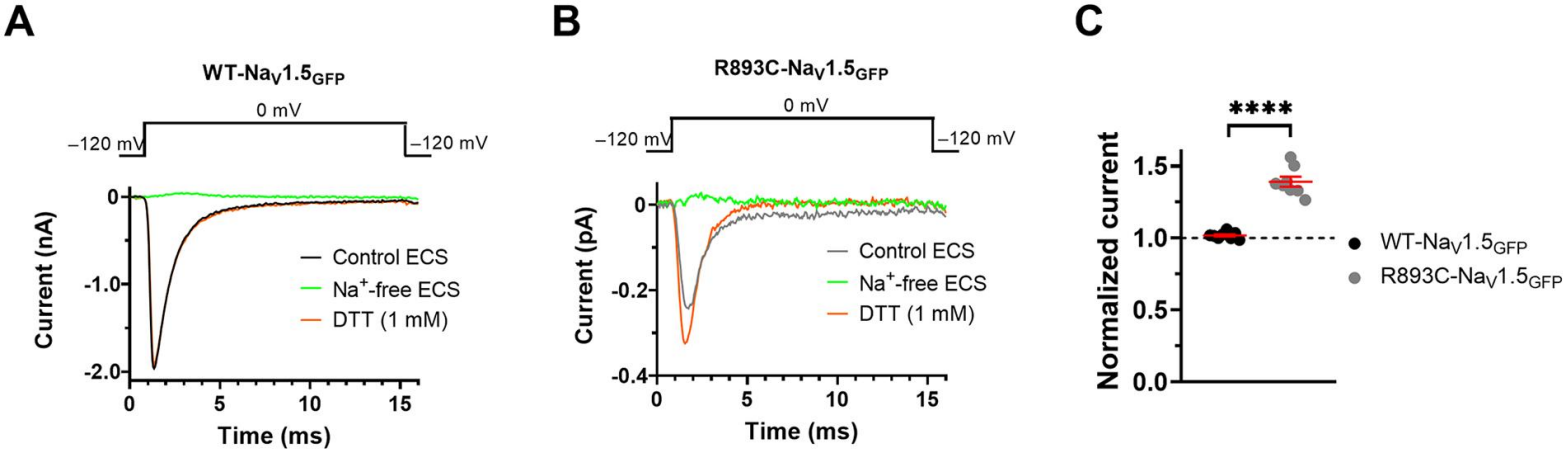
Effect of extracellularly applied reducing agent dithiothreitol on Na_V_1.5 currents. Representative whole-cell current traces obtained from CHO cells expressing either WT- **(A)** or R893C-Na_V_1.5_GFP_ **(B)** using a 15-ms long voltage step to 0 mV from a holding potential of −120 mV at a pulsing frequency of 0.2 Hz in control conditions (black), in the absence of extracellular Na^+^ (Na ^+^ -free ECS, green), and in the presence of 1 mM Dithiotreitol (DTT, orange), respectively. **(C)** Normalized current elicited by voltage steps to 0 mV was calculated as I/I_0_, where I_0_ is the peak current as control, and I is the peak current in the presence of 1 mM DTT after 3 min continuous perfusion. Symbols indicate individual data points for WT- (black) and R893C-Na_V_1.5_GFP_ (gray), respectively. Error bars indicate the mean ± SEM. ****, when *P* < 0.0001, t-test.

We hypothesized that the LOF current phenotype of *R893C* can be, at least partially, the consequence of the formation of disulfide bonds involving *893*C. To explore this scenario as the molecular mechanism of the current loss caused by the arginine to cysteine substitution, we recorded Na^+^ currents in the presence of the reducing agent dithiotreitol (DTT) at 1 mM. Voltage protocols shown in Figure 8 were used for recording Na^+^ currents in the continuous presence of 1 mM DTT, and its effect was expressed as normalized current calculated as I/I_0_, where I_0_ is the peak current at 0 mV prior to application of DTT and I is the peak current upon DTT application for 3 min. Exposure of CHO cells expressing WT-Na_V_1.5 to the reducing agent DTT for 3 min produced no functional changes in I_Na_ (Figure 8A), the normalized current was 1.02 ± 0.01 (*n* = 8). Importantly, DTT significantly enhanced the *R893C*-Na_V_1.5_GFP_ current; the normalized current was 1.39 ± 0.03 (*n* = 8) (*P* < 0.0001) (Figures 8B,C). These results suggest that *R893C* spontaneously form a disulfide bond with native cysteines in the vicinity of residue *893* that leads to the loss of the channel function. Moreover, reducing these disulfide bonds with DTT may help to restore the normal channel function in *R893C*-Na_V_1.5_GFP_.

### 3.7 The Na_V_1.5 Na^+^ channel functions critically depending on the *R893* residue

**Figure 9:**
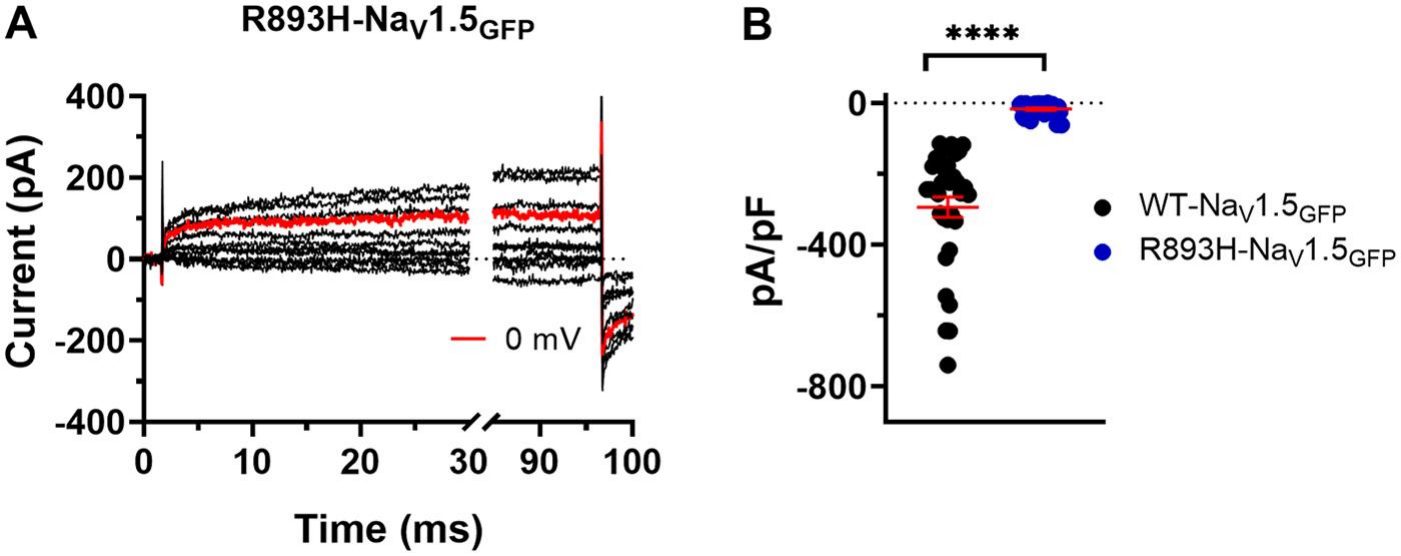
R893H mutation dramatically affects the normal function of Na_V_1.5. **(A)** Whole-cell currents were recorded at different test potentials ranging between −70 and 50 mV in 10-mV increments for R893H-Na_V_1.5_GFP_ channels expressing with transient transfection in CHO cells. **(B)** Current densities elicited at 0 mV. The peak current densities for a particular cell were defined as the average of current densities obtained for at least three depolarizing pulses repeated every 5 s in a sequence. Results are expressed as mean ± SEM. Symbols indicate individual data points obtained for the different Na_V_1.5 constructs. **** indicates significant differences when *P* < 0.0001 (t-test).

To further address the importance of the *R893* in the gating machinery of Na_V_1.5, we studied the functional consequence of the arginine to histidine mutation at the same site (*R893H*). If the conserved *R893* plays an important role in channel gating, then substitution with histidine, although having similar physicochemical properties like arginine, may also reduce the Na^+^ conductance, as well. In these experiments, we recorded whole cell Na^+^ currents at membrane potentials between −70 and +100 mV in CHO cells expressing *R893H*-Na_V_1.5_GFP_ (Figure 9A). When *R893H*-Na_V_1.5_GFP_ was expressed alone, very little current was observed. The current density was ∼3% of the WT-Na_V_1.5_GFP_ channels; at 0 mV current densities were (in pA/pF) 340.6 ± 36.2 (*n* = 31) for WT- Na_V_1.5_GFP_ and 17.3 ± 3.5 (*n* = 28) for *R893H*-Na_V_1.5_GFP_, respectively (*P* < 0.0001) (Figure 9B). We observed the same dramatic reduction in peak current densities by expressing *R893H*-Na_V_1.5_GFP_ in HEK cells (data not shown).

**Figure 10:**
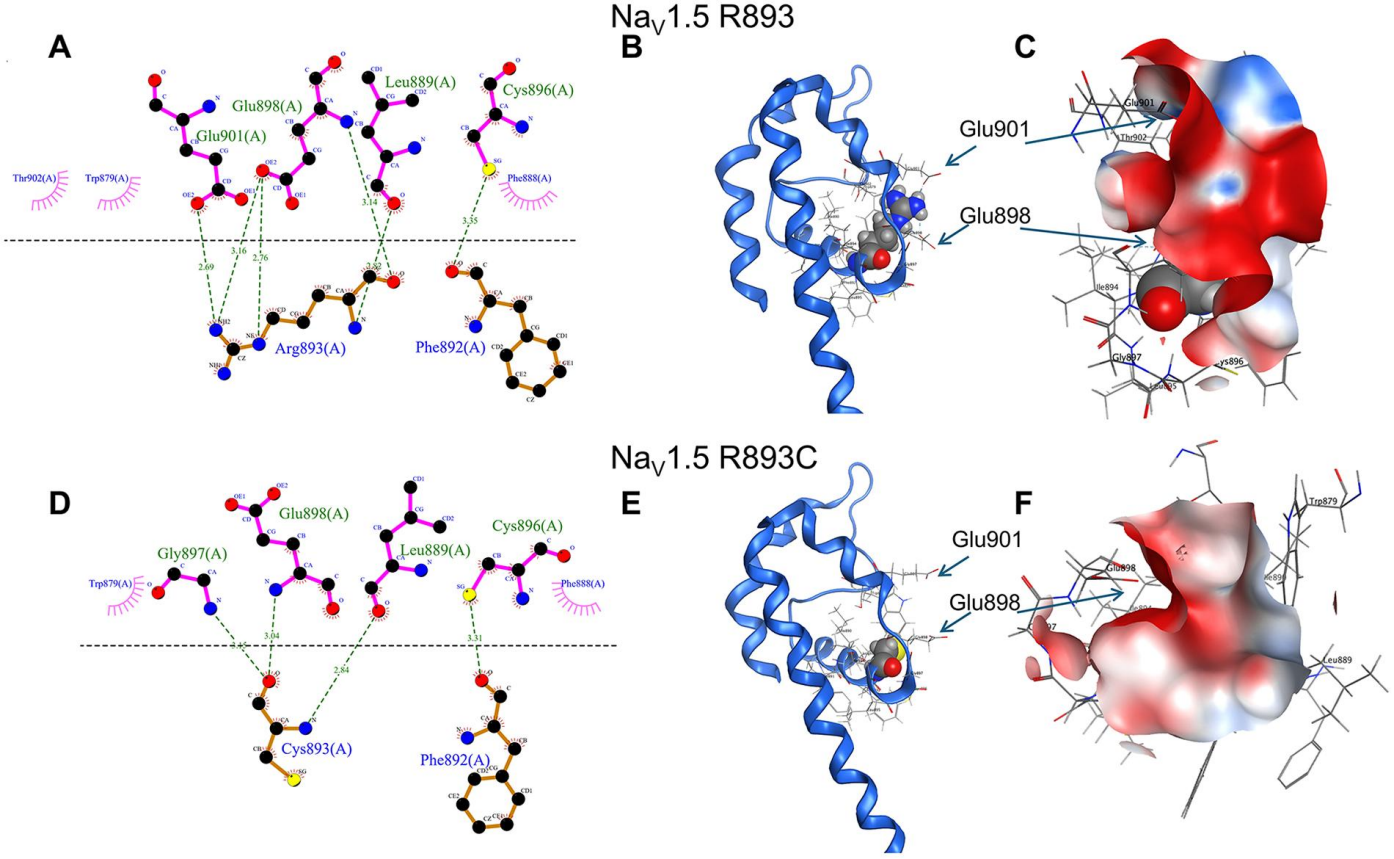
R893 is essential for the normal function of Na_V_1.5. Structural features are depicted in **(A–C)** for the WT-Na_V_1.5_GFP_, and **(D–F)** for the R893C-Na_V_1.5_GFP_. **(A,D)** 2D ligand interaction plots of R893 and R893C, respectively. Black, red, blue, and yellow dots indicate carbon, oxygen, nitrogen, and sulphur atoms, respectively. Magenta radial stripes around atoms and residues mark exposure to the environment. Hydrogen bonds are indicated by green dashed lines with the distance **(A)** of the H-bond. (**B** and **E**) 3D interactions in the DII S5-S6 loop (blue ribbon) of the wild-type R893 and R893C, respectively, with atoms highlighted as spheres. Atoms of residues in the vicinity are displayed as sticks; Arrows point to glutamates E898 and E901 facing towards the pore. In D), S322 is highlighted in dark green. **(C,F)** Snapshots of surface potential maps of residues neighboring 893 (R893 and R893C, respectively) were taken from the perspective of the channel pore. The range of the displayed surface potential map ranges from negative charge (red) to neutral (white) to positive charge (blue).

The findings above suggest that arginine at *893* is essential for the functional Na_V_1.5 channel activity. To better understand the structural implications of *R893*, we examined the cryo-EM structure of the human Na_V_1.5 channel (PDB: 7DTC). Indeed, *R893* appears to play a critical role in stabilizing the pore helix by forming three hydrogen bonds with the side chains of glutamate residues E898 and E901 (Figure 10A), thereby coordinating their negatively charged side chains towards the pore (Figure 10B). This coordination likely helps maintain a negatively charged electrostatic environment (Figure 10C) that is favorable for Na⁺ conductance. In contrast, the *R893C* mutant lacks the conservative bulky arginine side chain necessary for this coordination, resulting in a disruption of these interactions (Figures 10D,E). Consequently, the negative charge density in this region is significantly reduced (Figure 10F), which may impair Na⁺ ion permeation through the pore.

## 4 Discussion

In this study, we functionally characterized an *SCN5A* mutation (c.2677C > T) associated with a peculiar clinical phenotype in a Hungarian proband, a 41-year-old male patient diagnosed with BrS. A marked variability in the clinical presentation ranging from the absence of any symptom to SCD can be observed in individuals carrying *SCN5A* mutations (27). Due to its heterogeneity, the functional characterization of any mutation in patients with BrS may provide further contribution to the understanding of the disease.

The c.2677 C > T nucleotide change causes an arginine-to-cysteine substitution at position *893* (p.*R893C*), localized in the S5-S6 extracellular linker in DII (Figure 4). The region of our interest is a hotspot for several mutations causing BrS (6). For example, e.g., R878C was examined in a Chinese family (28) that, and a decreased Na^+^ conductance was reported without dominant negative effect on the WT channels. Another mutation in the same position, R878 K (28), also resulted in the loss of the Na^+^ current, highlighting the importance of the highly conserved Arg residues in the pore region. We hypothesized that the *R893C* heterozygous mutation is pathogenic based on the followings: (1) sequence alignment demonstrated that *R893* is highly conserved in human Na_V_ channel isoforms and across mammal species (Figures 5A,B) suggesting that *R893* is likely to be essential for normal channel function; (2) algorithms developed to predict the effect of missense changes on protein structure and function (SIFT, PolyPhen-2, Align-GVGD) all suggested that *R893C* variant is likely to be disruptive; 3) *SCN5A* c.2677C > T was absent in a large cohort (6,500) of healthy individuals (ClinVar and Ensemble database) and was not found in the Human Gene Mutation Database (HGMD) (20), Ensembl, HapMap, and 1,000 genomes projects, thereby excluding a single nucleotide polymorphism. Our study confirmed the hypothesis, the *R893C* Na_V_1.5 displayed significant loss of channel function, i.e., dramatically decreased current density. The LOF phenotype reported in our study is in a good agreement with previous findings i.e., the current density of *R893C* is approximately 5%–10% of WT Na_V_1.5 expressing HEK cells (12, 14). To provide a detailed biophysical characterization of *R893C-*Na_V_1.5 and shed light on the molecular mechanism of the significant Na^+^ current loss we followed the principles in the literature regarding the characterization of genotype-phenotype relationships in BrS (29–31).

For strategic purposes we used Na_V_1.5 constructs carrying GFP-tag at the N-terminus that allowed us to track the expression level of the different Na_V_1.5 constructs in the membrane or the localization of the proteins in the cell. We clearly showed that the GFP-tag does not modify the gating behavior of Na_V_1.5 (Supplementary Figure S3) similarly to the observations for the human heart Na^+^ channel (hH1) protein (32).

We argue that the small I_Na_ recorded in *R893C-*Na_V_1.5 expressing cells is conducted by the *R893C-*Na_V_1.5 channels, rather than the endogenous Na_V_ channels know to be expressed in CHO (22), as follows: (1) We observed a very low background Na^+^ current in non-transfected CHO (Supplementary Figure S5); (2) The *R893C*-Na_V_1.5 current retained its TTX resistant phenotype (Figure 4B) characteristic of Na_V_1.5 [IC_50_ = 5.7 µM (23, 24),]. Contrary to this, endogenous Na^+^ channels in CHO can be completely inhibited by nanomolar concentrations of TTX (22). In addition, *R893C*-Na_V_1.5 channels are expressed in membrane as reported by fluorescence confocal microscopy and SDS-PAGE (Supplementary Figure S6 and S7). Based on these we conclude that the Na^+^ current measured in *R893C-*Na_V_1.5 expressing cells is the consequence of the expression of non- or reduced function of Na_V_1.5 channels. Moreover, changes in the biophysical parameters of the currents discussed below are due to altered intrinsic properties of the *R893C-*Na_V_1.5 channel.

Most Na_V_1.5 mutations associated with BrS are LOF mutations and are commonly due to disruption of activation or impaired recovery from inactivation (33, 34). We also observed slower activation kinetics of I_Na_ current for *R893C* channels, together with a depolarizing shift in the voltage dependence of steady-state activation with a shallower slope (Figures 5A,B). Moreover, the activation kinetics showed similar voltage sensitivity both for the WT and the *R893C* variants (Supplementary Figure S8). The slowing of the activation kinetics maintained fast inactivation kinetics (Figure 5A) may contribute partially to the decreased Na^+^ current density since channels are more likely to inactivate before they the current would reach its maximum at a given membrane potential (35). This, together with the depolarizing shift in the voltage-dependence of steady-state activation of the *R893C* variant may reduce the currents at physiologically relevant membrane potentials.

Surprisingly, the *R893C* mutation also caused a 10-mV shift towards more positive membrane potentials in the voltage dependence of steady-state inactivation (Figure 5C). Similarly, a relatively large depolarizing shift in the voltage dependence of steady state inactivation was observed in the LOF mutant T1857I-Na_V_1.5, that is linked to a family with a catecholaminergic polymorphic ventricular tachycardia (CPVT)-like phenotype characterized by a strong family history of SCD (36). The depolarizing shift in the voltage dependence of steady-state inactivation increases the number of available channels that can be active, that, therefore, may compensate the reduced current density, as it widens the window of potentials (i.e., window current) over which a fraction of sodium channels can be activated, but not inactivated i.e., functional.

To mimic the heterozygous condition of the patient and test a potential dominant-negative effect of the mutation, we co-transfected WT- and *R893C*-Na_V_1.5_GFP_ at 1:1 molar ratio (using 1-1 ug DNA) into CHO cells and determined the peak current amplitudes and kinetic parameters of the whole-cell Na^+^ currents (Figure 6). Our analysis showed that the presence of the *R893C*-Na_V_1.5_GFP_ channels in the membrane did not exert a dominant negative phenotypic effect. Our results are in good agreement with earlier reports, e.g., T187I, D356N, K1578fs/52, and R1623X, that were similarly linked to BrS, SSS and atrioventricular block, also lacked a dominant-negative effect (37). Furthermore, confocal microscope imaging yielded similar plasma membrane expression of both WT-Na_V_1.5_GFP_ and *R893C*-Na_V_1.5_GFP_ channels (Supplementary Figures S6 and S7). There are, however, limitations for the interpretation of the “mixed” co-expression data, where WT- and *R893C*-Na_V_1.5_GFP_ were expressed simultaneously, for mimicking heterozygosity. We assumed that both constructs have equally expressed. Co-expression of wild-type and mutant Na_V_1.5 channels were reported to form dimers, and due to their interaction, coupled gating of voltage-gated Na^+^ channels may result in a dominant negative effect. Nevertheless, we have not seen a dominant negative effect, i.e., the gating and permeability changes reported in our paper may not couple like in case of L325R (38), even if dimers are formed.

Sodium channel blocker procainamide is frequently used to unmask the Brugada ECG whenever the disease is suspected (25, 26). Accordingly, the transformation of a normal ECG pattern to a coved ST elevation in response to the procainamide administration is specific for BrS (39), as observed in the proband (13). We observed almost complete inhibition of I_Na_ by 1 mM procainamide in all variants (Figure 7). This finding supports that the mutant channel retained its procainamide sensitivity which correlates well with the clinical findings during BrS induction.

We found that extracellularly applied DTT at 1 mM partially restored the normal function of Na_V_1.5 in the *R893C*-Na_V_1.5_GFP_ variant (Figure 8). Based on the domain structure and the available cryo-EM structure of Na_V_1.5 (17, 40), we propose the idea that the cysteine at *893* could form disulphide bridges with native cysteine residues in the close vicinity of *R893C*, e.g., C896. Other cysteines in the same linker, such as C906 and C915, and more distant cysteines located in other domains (e.g., C373 in DI, C1272 in DIII, or C1703 in DIV) are too far away to form plausible bonds with *R893C*. Based on these we propose that the second component of the reduction in the I_Na_ in the *R893C*-Na_V_1.5 in may be attributed to the formation of disulphide bridges that disrupt Na^+^ permeation (see below).

To further test the critical role of the conserved R893, we studied the electrophysiological properties of R893H-Na_V_1.5_GFP_ expressed in CHO cells. This mutation is also a missense pathogenic mutation that was identified in patients with BrS (6, 41). Patch-clamp recordings revealed that R893H-Na_V_1.5GFP failed to show detectable macroscopic I_Na_ (Figure 9).

Our findings complement and confirm previous reports of highly conserved arginine residues at different positions in Na_V_-s that are critical for channel function (6). For example, R878 is similarly highly conserved, and the replacements of either R878C or R878 K failed to produce any detectable I_Na_ (26).

Closer inspection of the wild-type and mutant structures revealed additional mechanistic insights for the decrease in the I_Na_ for the R893 mutants. The positively charged bulky arginine *R893* in the WT channel appears to stabilize the pore loop by forming three hydrogen bonds with the side chains of two glutamates, E898, and E901 (Figure 10A). By sterically coordinating the negatively charged glutamate sidechains towards the pore (Figure 10B), these interactions likely maintain a negatively charged electrostatic environment favorable for Na⁺ conductance (Figure 10C). However, the energy-minimized structure indicates that sterically more favorable interactions might occur between *R893C* and the backbones of adjacent residues such as L889, G897, E898 (Figure 10D). Therefore, the *R893C* mutant lacks the ability to coordinate E898, and E901 (Figure 10E), leading to a significant reduction in negative charge density in this region (Figure 10F), which may impair ion permeation. A similar disruption was observed in the *R893H* mutant, which lacks sufficient size and charge to stabilize E901 effectively (not shown). These structural predictions corroborate our electrophysiological experimental data and support the notion that *R893* is essential for channel function and plausibly explain its evolutionary conservation. A similar mechanism has already been highlighted for the R878C mutant in Na_V_1.5 which failed to show detectable I_Na_, and proposed to be associated with familiar sick sinus syndrome (SSS) in a Chinese family (28).

Moreover, Arg residues behind the SF, such as R367, R383, R878, *R893*, form salt bridges with negatively charged residues (42). Breaking critical salt bridges in these Arg mutations can induce conformational changes of the SF and then reduce the ion permeability (42). Due to their location, these Arg residues may also play key roles in fast inactivation, persistent current, membrane protein orientation, protein-lipid interactions and protein-protein interactions (43–45). Disruption of these mechanisms may also contribute to the pathogenic nature of the aforementioned Arg mutations. Since *R893* directly forms salt bridges with two glutamate residues, E898 and E901 in DII (42), the *R893C* mutation may disrupt these critical interactions adjacent to the SF thereby inducing conformational changes in the SF region and consequently reduce Na^+^ permeability. In harmony with these both *R893C* and E901 K have been associated with BrS (6). Similarly to this, R878 forms a salt bridge with D1430 in DIII from the neighboring repeat (42). In summary, a third mechanism for the reduction in the I_Na_ in the *R893C*-Na_V_1.5 may be attributed to the disruption of the interaction network required for Na^+^ conductance, that is maintained by a critical Arg (R893) in the WT channel.

Based on our results, we argue that the abnormal Na^+^ homeostasis, caused by the loss of function of *R893* mutations, plays a critical role in creating an arrhythmia-prone substrate as well as in generating a trigger for the rhythm disorder. Reduced sodium current decreases AP's upstroke velocity causing longer AP, leading to atrial and ventricular conduction slowing accompanied by prolongation of PR- and QRS-intervals on the ECG. According to one possible scenario, the sodium current reduction may cause an endo-epicardial potential gradient underlying the right precordial ST-segment elevation that may further exacerbate right ventricular activation delay and subsequent ECG changes (41, 46). This endo-epicardial potential gradient and the consequential repolarization heterogeneity may act as the electrophysiological substrate to ventricular arrhythmias. Moreover, the decreased Na^+^ conductance makes the AP more vulnerable to early and delayed afterdepolarizations (EAD and DAD): if these afterdepolarizations reach the activation threshold, a new AP can be triggered before the first one ended, which can cause an “R on T” phenomenon, inducing polymorphic VT.

In summary, we have characterized a Na_V_1.5 pore mutation, *R893C*, in a male patient diagnosed with BrS. This mutation causes an evident reduction in I_Na_, which originates from multiple components including alerted time- and voltage-depend gating of the mutant channels; disulphide bridge formation between *R893C* and an adjacent cysteine residue; and a disrupted interaction network of amino acid side chains required for Na^+^ conductance. The mutation-dependent effects create the conditions for the pathophysiological manifestations observed in the patient. Although the changes in the function of the mutant channel are significant, we cannot exclude that the functional consequences of *R893C* mutation in native myocytes are different from those observed in our CHO cell experimental model, due to different regulatory factors. We strongly believe that our results hold the promise that correlations among channel structure, functional effects of mutations and clinical studies can lead to better understanding of arrythmia-related channelopathies, such as BrS. Moreover, understanding the structure-function relationship of Na_v_1.5 may improve novel gene therapy and shed new light on exploiting efficient medication for *SCN5A* channelopathies.

## Data Availability

The original contributions presented in the study are included in the article/Supplementary Material, further inquiries can be directed to the corresponding author.
