# Supplementary material for: Characterization of the R893C NaV1.5 mutation in Brugada syndrome
Source: Front Cardiovasc Med. 2026 Feb 19;13:1726536. doi: 10.3389/fcvm.2026.1726536 (PMC12960491; doi:10.3389/fcvm.2026.1726536)
Supplement: Supplementary file 1 [file Datasheet1.pdf]

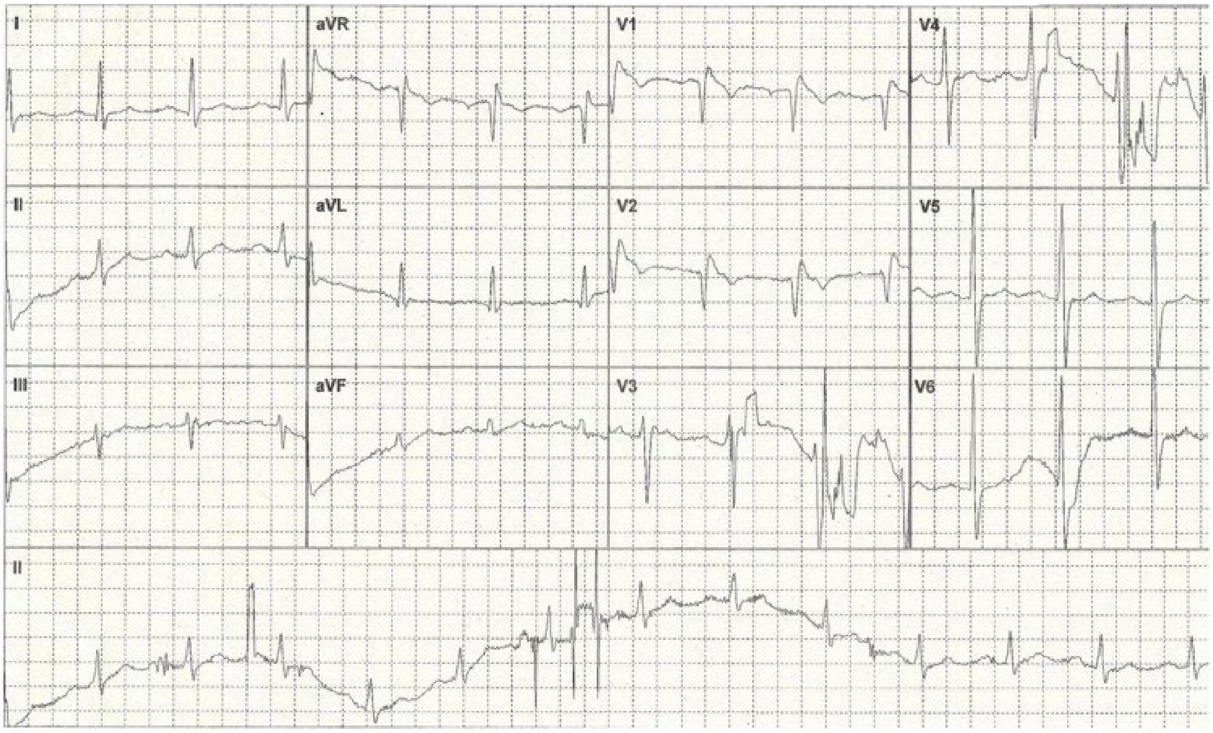

A

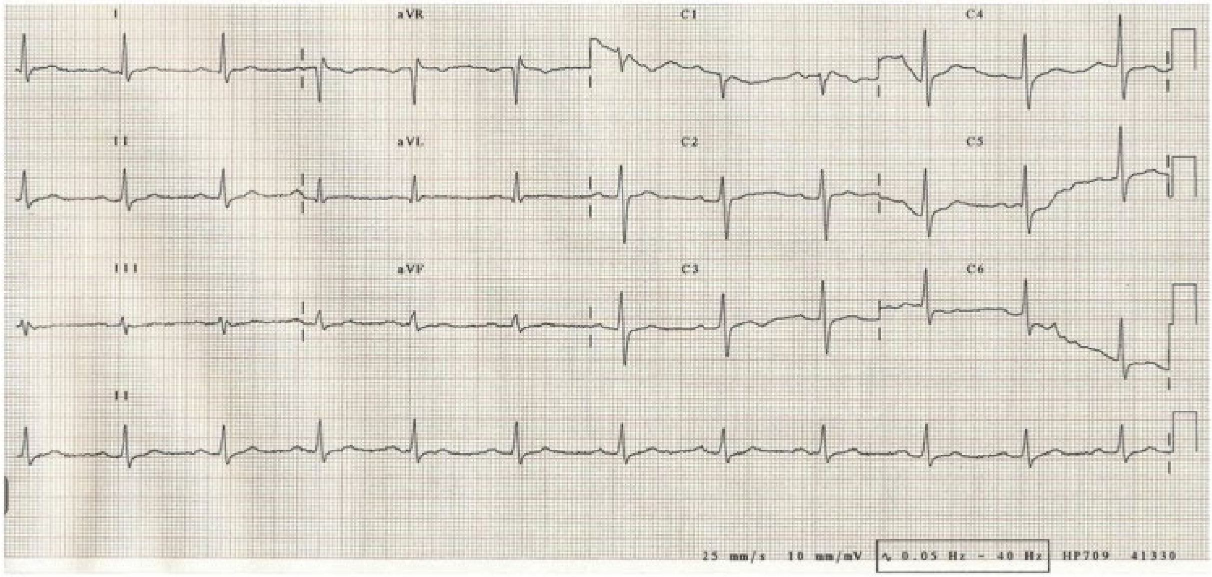

B

Supplementary Figure 1. (**A**) A transtelephonic ECG (TTECG) sent by the ambulance team, showing type 1 Brugada-syndrome pattern, (**B**) Resting ECG of the proband after arrival, the BrS pattern resolved.

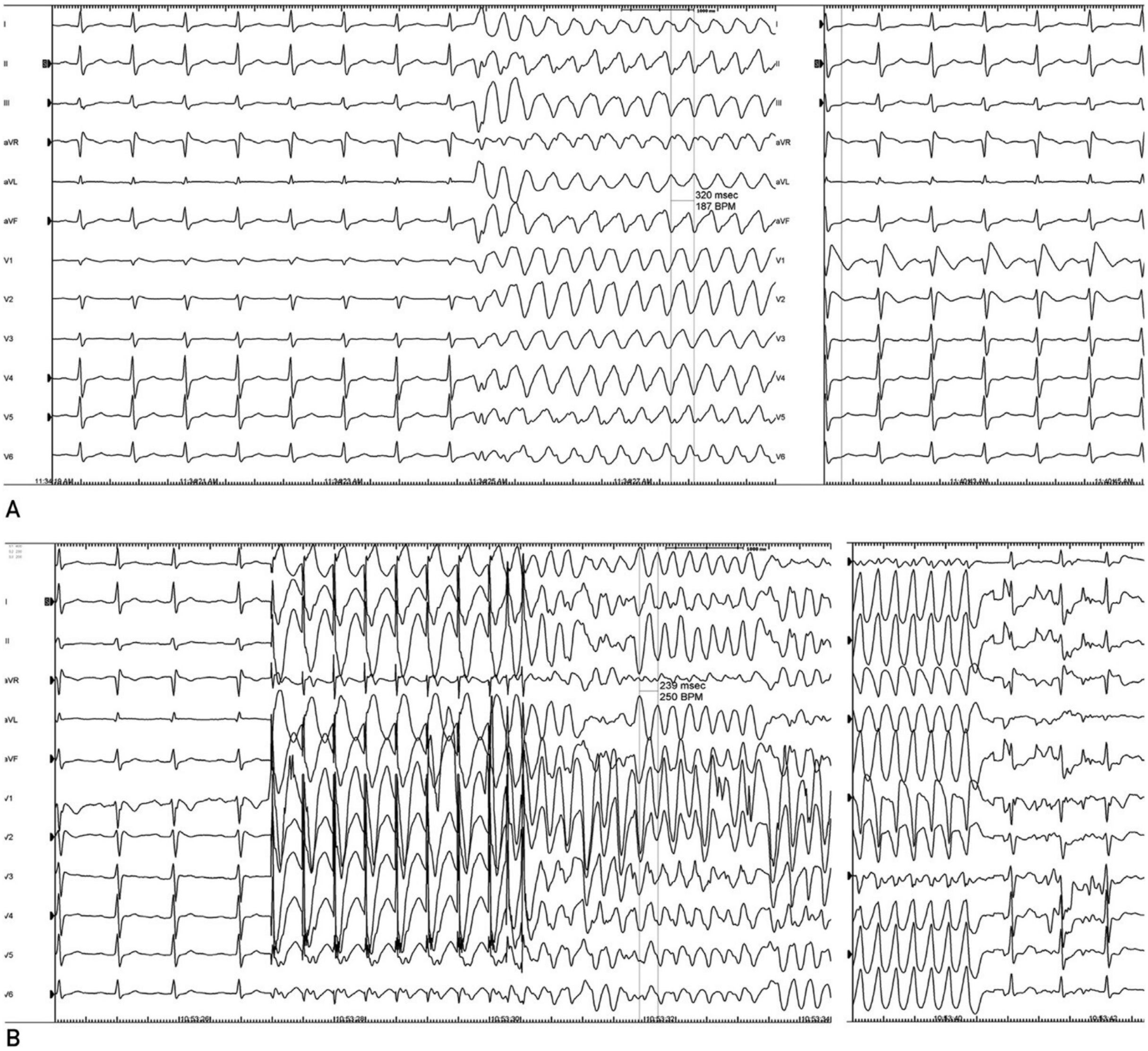

Supplementary Figure 2. Normal 12-lead surface ECG during an invasive electrophysiology study. (**A**) Slow intravenous administration of Procainamide (400 mg) induced a fast (187 beats per minute − BPM) ventricular tachycardia (VT) at 11:34:25 AM, after termination type 1 BrS pattern appeared in V1-2 leads, (**B**) Programmed electrical stimulation of the right ventricle apex with 2 extrastimuli at 10:53:30 AM caused fast (250 BPM) polymorphic VT.

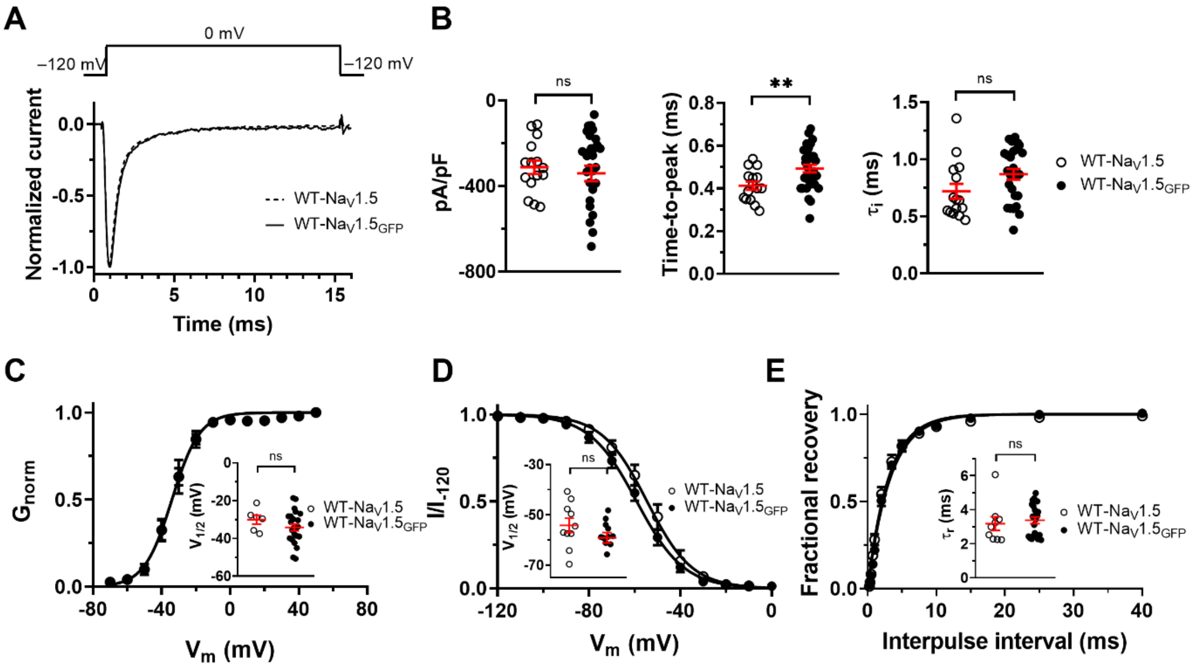

Supplementary Figure 3. (A) Representative whole-cell current traces were recorded for wild type $Na_V1.5$ (dashed line) and WT-$Na_V1.5_{GFP}$ channels (solid line) expressed in CHO cells using the voltage protocol shown above the raw current traces. **(B)** Left: current densities measured at 0 mV. Sodium current amplitude was normalized to the cell capacitance to obtain current density values. Middle: kinetics of activation was characterized by determining the time-to-peak of I_{Na} shown in (A). Right: time constant of inactivation (τ_i) was determined by fitting a single exponential function to the decaying part of the currents shown in (A). **(C)** The voltage-dependence of steady-state activation was determined from the peak currents at test potentials V_m ranging from –70 mV to +50 mV in 10-mV increments every 15 s. Inset: points indicate individual data points of the midpoint voltages ($V_{1/2}$) of steady-state activation. (**D**) Steady-state inactivation was determined using the voltage protocol described in Materials and Methods. Inset: data points indicate individual data points of the midpoint voltages ($V_{1/2}$) of steady-state inactivation. The superimposed solid lines show the best fit Boltzmann function for (C) and (D), respectively. **(E)** Kinetics of recovery from inactivation was studied using a two-pulse protocol with increasing interpulse time, (ipi, from 0.25 to 40 ms) between the pulses (see Materials and Methods for details). Data points presented as fractional recovery and plotted as a function of ipi. The time constant of recovery (τ_r) was determined by fitting the data points with a single exponential function, the superimposed solid lines show the best fits. Inset: Symbols indicate individual data points obtained for WT-$Na_V1.5$ (filled circles) and WT-$Na_V1.5_{GFP}$ (empty circles), whilst error bars indicate the mean ± SEM of the investigated parameter throughout the Figure. Differences were considered significant (**, when $P < 0.01$), ns indicates non-significant differences when $P > 0.05$ compared to WT-$Na_V1.5_{GFP}$ (*t* test).

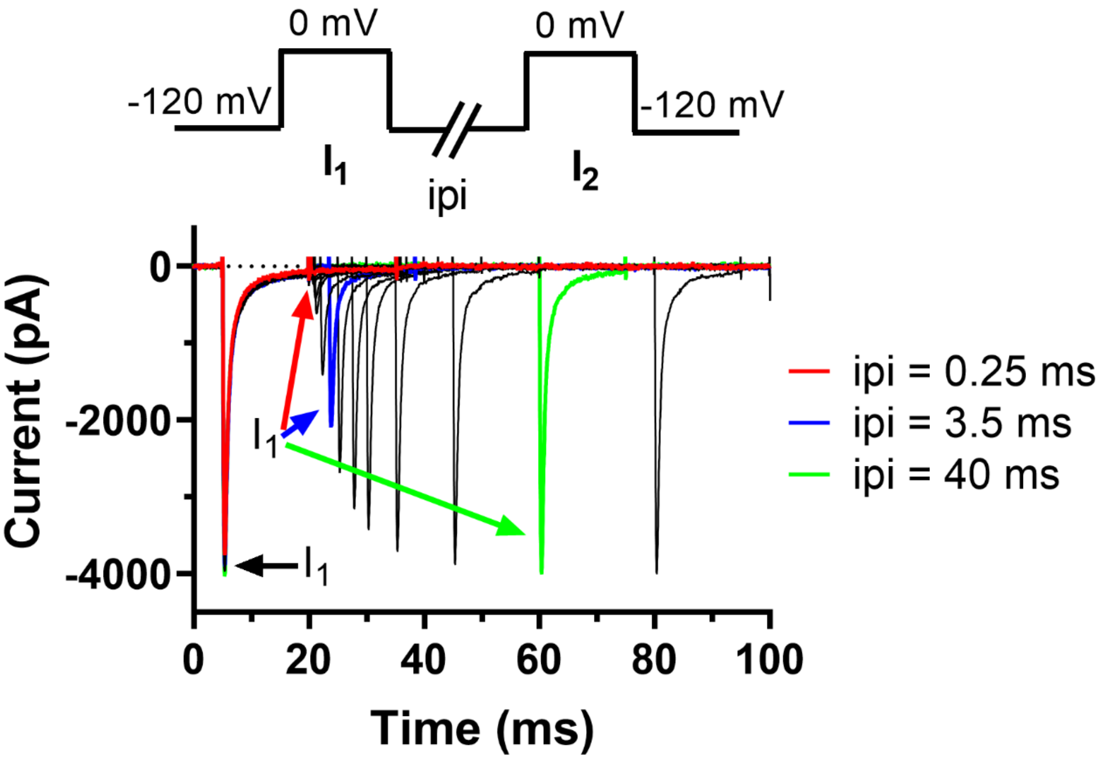

Supplementary Figure 4. For measuring the kinetics of recovery from inactivation, pairs of depolarizing pulses were delivered from the holding potential of −120 to 0 mV for 10 ms (I_1, black arrow) (see the voltage protocol above the raw current traces). Currents recorded during the second pulses (I_2) are shown at various ipi at −120 mV holding. Traces recorded with ipi of 0.25, 3.5 ms, and 40 ms indicated by arrows and highlighted in red, blue, and green, respectively. The fractional recovery was calculated as I_2/I_1, where I_2 and I_1 are the peak currents during the second and first pulse, respectively.

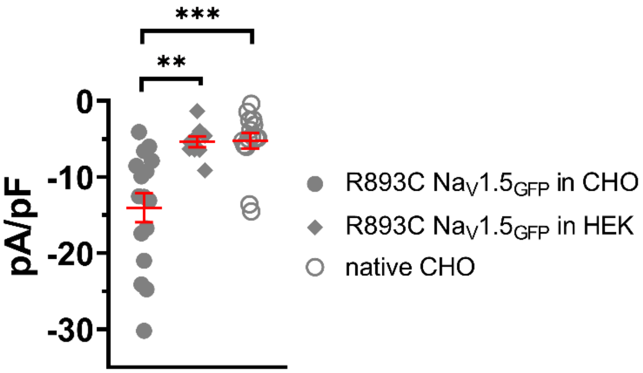

Supplementary Figure 5. Current densities measured at 0 mV. Cells were held at −120 mV and depolarized to 0 mV for 15 ms every 5 s before returning to the holding potential. I_{Na} amplitude was normalized to the cell capacitance to obtain current density values at each test potential for R893C expressed in CHO (filled circles), R893C expressed in HEK (diamond), and native CHO (empty circles). Values are expressed as mean ± SEM of 9-16 cells is reported. Symbols indicate individual data points. Differences among groups (ANOVA) were considered significant (**, when $P < 0.01$ and ***, when $P < 0.005$) compared to WT-Na$_V$1.5$_{GFP}$ (Tukey's post hoc).

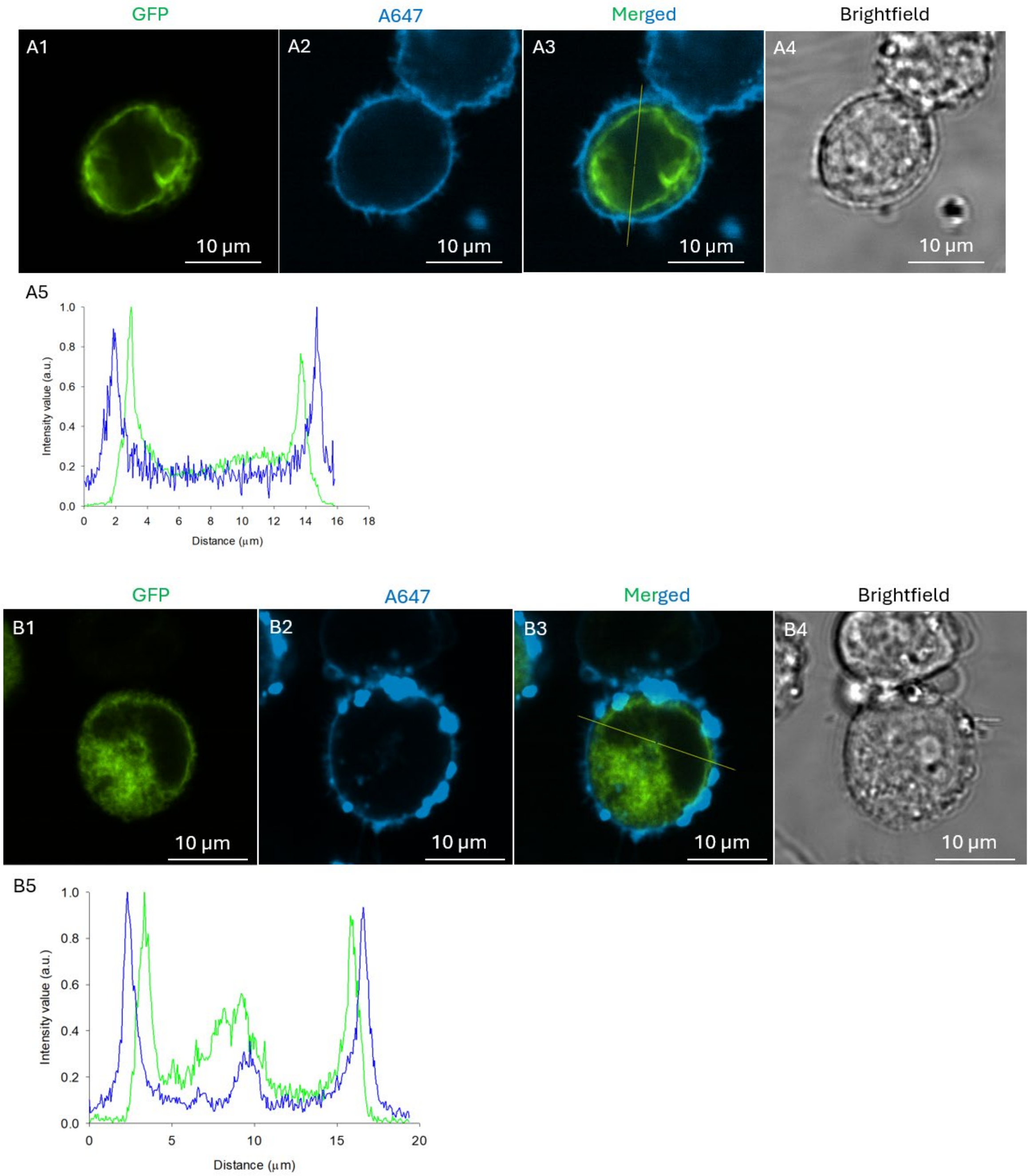

Supplementary Figure 6. Confocal microscopic images of WT-Na$_V$1.5$_{GFP}$ (**A**) and R893C Na$_V$1.5$_{GFP}$ (**B**) transiently transfected in CHO cells. Images demonstrate the GFP fluorescence signal of WT-Na$_V$1.5$_{GFP}$ or R893C Na$_V$1.5$_{GFP}$ (A1, B1, green), the Alexa Fluor™ 647 NHS Ester fluorescence signal of the plasma membrane (A2, B2, blue) of the same cells. (A3, B3) Merged confocal fluorescence microscopy images. (A4, B4) Brightfield images of GFP-tagged WT or R893C Na$_V$1.5 transfected cells. Scales bars are 10 µm for each panel. The normalized pixel-by-pixel intensity profile analysis of the two fluorophores of linear locations analysed with different images for described WT (A5) and R893C Na$_V$1.5 (B5). The intensity profile analysis indicates the similarity between WT and R893C Na$_V$1.5.

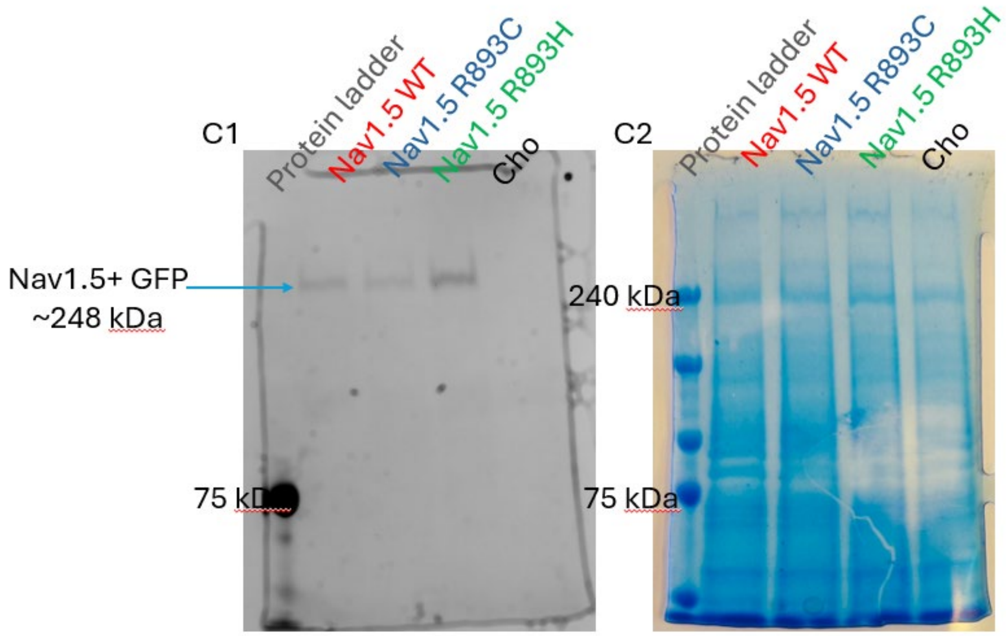

Supplementary Figure 7. SDS-PAGE analysis of GFP signals emitted by $Na_V1.5$ variants extracted from membrane fractions of transiently transfected CHO cells. (C1) SDS–polyacrylamide gel analysis of $Na_V1.5$ constructs using Alpha Innotech gel documentation system for the detection of the GFP signal. The protein ladder (grey) indicates the size of $Na_V1.5$ proteins, the bands show the GFP signal for WT-$Na_V1.5_{GFP}$ (red), R893C $Na_V1.5_{GFP}$ (blue), R893H $Na_V1.5_{GFP}$ (green), and non-transfected CHO cells (black). The size of the GFP-tagged Nav1.5 is about 248 kDa. No fluorescence emission was detected for the non-transfected CHO cells. (C2) SDS–PAGE analysis of CHO cells expressing GFP fluorescent $Na_V1.5$ variants. Cells were lysed by boiling in sample buffer and processed for 10–20% gradient SDS–PAGE stained with Coomassie blue.

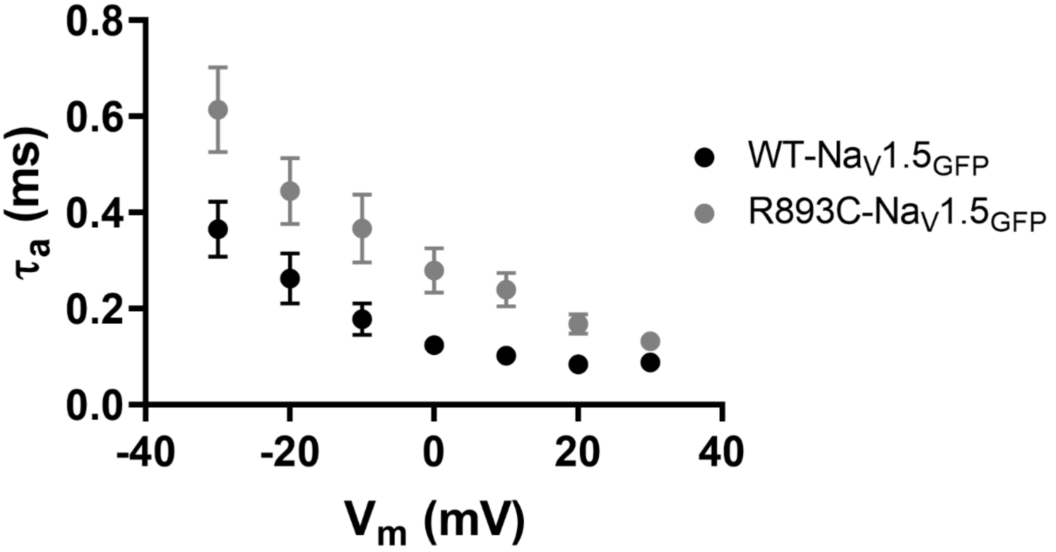

Supplementary Figure 8. The voltage dependence of the kinetics of activation. To analyze the activation kinetics Na^+ current traces were fitted with an exponential function rising to the maximum using $I(t) = I_a\left(1 - e^{(-t/\tau_i)}\right)^4 + C$, I_a is the amplitude of the activating curve component, τ_a is the activation time constant of the current, and C is the amplitude of the non-activating current component. The τ_a for a particular cell was determined as the average ± SEM of τ_a values obtained for 4-5 depolarizing pulses repeated at every 5 s.
